# Influences on the dietary intakes of preschool children: a systematic scoping review

**DOI:** 10.1186/s12966-022-01254-8

**Published:** 2022-02-22

**Authors:** M. Jarman, K. Edwards, J. Blissett

**Affiliations:** School of Psychology, College of Health and Life Sciences, and Institute of Health and Neurodevelopment, Aston University, Birmingham, UK

**Keywords:** Diet, Scoping review, Preschool children, Ecological systems theory

## Abstract

**Background:**

Better diet quality of preschool children is associated with many important health outcomes, but there is significant room for improvement in many children’s dietary intakes. The determinants of children’s dietary intakes are complex and whole systems approaches may be effective tools for changing dietary intake. Collation of all the evidence available on determinants of preschool children’s dietary intake is necessary to ‘map’ the whole system of influence. Therefore, this systematic scoping review of available literature on determinants of dietary intakes in preschool children was undertaken.

**Methods:**

The Joanna Briggs Institute methods for conducting a systematic scoping review were followed. Articles published since 2000 which assessed influences on the dietary intakes of preschool children were identified, yielding a total of 246 papers. Studies of children with clinical conditions (excluding obesity), or those conducted in middle and low-income countries were excluded, due to the different systems of influence in these populations. Data were extracted and information synthesised based on ecological level (child, parent, household, childcare, or wider determinants).

**Results:**

Most articles focused on influences at the parental level (*n* = 118, 48%), followed by those at the child level (*n* = 73, 30%). Most of the studies were of cross-sectional design (*n* = 109, 44%). Whilst many studies considered influences at multiple ecological levels (*n* = 63, 26%) few analyses determined interactions between factors in their relationship with children’s dietary intakes, which is needed going forward using systems methods.

**Conclusion:**

A wealth of evidence exists examining influences on the dietary intakes of preschool children and this information would benefit from analysis using a systems thinking approach in order to assess effective levers for intervention and what works, for whom, under what circumstances.

**Supplementary Information:**

The online version contains supplementary material available at 10.1186/s12966-022-01254-8.

## Introduction

The establishment of a healthy diet in the preschool years (typically aged 2–5 years) is crucial for the short- and long-term health of an individual. Across the world, a good quality diet tends to consist of frequent consumption of fruits, vegetables, wholegrains and lean sources of protein and dairy, as well as infrequent consumption of foods high in sugar, salt and/or fat with low nutrient density. In the short term, better quality diets of preschool children have been associated with better cognitive development [1] and lower risk of overweight and obesity [2, 3]. In the longer term, diet quality tends to track from preschool to adolescence and beyond [4] thus setting individuals on a lifelong trajectory of risk of poorer or better health.

Across high-income countries, national level surveys and studies have indicated that the quality of preschool children’s diets have room for improvement. A recent report from the National Diet and Nutrition Survey (NDNS) in the UK showed that preschool children (aged 1.5–3 years) obtained ~ 11% of their total daily energy intake from added sugar, which is more than double the recommended contribution [5]. In addition, this age group were only consuming around 2 servings of fruits and vegetables a day. In the USA, the Feeding Infants and Toddlers survey (FITs) showed that the most commonly reported vegetable consumed by 2–4 year olds was fried potato and that 27% of children did not consume a single portion of vegetables on the day of reporting [6]. Furthermore, in Canada a recent study of adherence to national dietary guidelines of 3-year-olds showed that only 38% of children met the recommendations for intake of milk or alternatives and 13% met the recommendation for intake of grains [7].

Due to the variation in the quality of preschool children’s diets and its impact on health, many studies have examined the key influences on children’s food intakes in order to identify target levers for intervention. It is undisputed that determinants of children’s food intakes are complex and interventions aimed at improving children’s diet quality have commonly included a number of targets [8–10]. However, the most effective approaches have yet to be discovered, as evidenced by limited sustainability of their impacts [8–10]. In response to the lack of long-term effective interventions in improving childhood diet and obesity rates, there has been a shift in the methods used to assess influences on children’s diet quality. Researchers and policy makers now consider a whole systems approach [11, 12], which incorporates mapping of the complex, interacting factors which result in dietary outcomes, tailored to the specific context, including multiple dynamic feedback loops and multiple stakeholders within the system. Whole systems models have heterogenous elements which interact to produce an outcome which differs from the individual effects of these elements on outcome, and which produces effects which may vary across time and changing circumstance [13]. Systems thinking, particularly when combined with simulations, can facilitate understanding of complex health problems and also guide strategic investment in policy and practice with lowest cost and greatest benefits [14].

Whole systems approaches are powerful tools for changing complex behaviours, but to be used effectively, the first stage requires collation of all the evidence available on determinants of the health outcome in question, in order to ‘map’ the whole system of influence. To date there has been no comprehensive literature review of influences on preschool children’s diets. Therefore, a systematic scoping review of the literature available on determinants of food intakes in preschool children was undertaken. A systematic scoping review approach was adopted because this is the most appropriate method for synthesising evidence to identify key characteristics or factors related to a broad topic or concept, where studies have adopted multiple study designs, and where the ultimate aim is to explore the range and extent of research to bring together important concepts, results and gaps in the evidence [15–17]. We have applied an ecological systems model to structure the evidence. Ecological systems models are well established in children’s eating behaviour literature; for example, Davison and Birch (2001) [18] described the use of this approach to understand child overweight, in which child factors, such as gender or age, are embedded in the family, which are in turn situated in communities and larger societal influences. Here, we use an ecological systems approach to structure the evidence according to the level of influence on children’s diets, at the individual (child) level, parent level, household level, childcare centre level or wider societal level.

## Methods

We followed the methods as outlined by the Joanna Briggs Institute for conducting a systematic scoping review [15, 16] and completed the Preferred Reporting Items for Systematic Reviews and Meta-Analyses (PRISMA-ScR) extension for scoping reviews checklist [19] (Additional file 1).

### Search strategy

The full a priori search protocol is shown in Additional file 2. The databases Medline, Embase, Scopus, Web of Knowledge, PsychINFO and the Cochrane Library were searched by two researchers (MJ and KE) in May 2019 using the search terms shown in Additional file 2. The databases OpenAire, EThOS, and Proquest, were also searched to identify grey literature and/or theses. In addition, bibliographies of relevant review articles were screened to identify any additional articles to include in the selection process. The search was rerun in May 2021 to identify any new articles which had been published between the initial search and submission of the article (*n* = 32 included for review).

### Study selection

Articles were excluded from the process according to the inclusion/exclusion criteria shown in Table 1. The study team discussed the inclusion/exclusion criteria at length, it was important to be inclusive enough to address the research question whilst having limits in order to give clarity to the application of the results to a specific population. To remove some of these exclusion criteria would have rendered the review too large and difficult to present and interpret. In particular the researchers narrowed the study selection to preschool children (but not infants), without clinical conditions, living in developed countries, with a dietary intake variable as an outcome variable, and those published before 2000. The researchers defined included outcome variables as ‘any assessment of food, drink or energy intake’, even if a specific behaviour was being assessed ‘e.g. eating in the absence of hunger’. In order to refine the articles returned in the initial searches the list of titles was divided equally between the researchers (MJ and KE) for screening. Once the initial title review was complete the remaining articles were imported into ENDNOTE to allow for duplicate removal. This was followed by a screening of the abstracts of the remaining articles, again the list of abstracts was divided between the two researchers for screening and a random sample of 20 abstracts were screened by both researchers to ensure consistency in the inclusion/exclusion process. There were no discrepancies between the researchers in the consistency check. Once articles had been refined by undergoing title and abstract review the remaining full text articles were read by both researchers and a final list of included manuscripts was compiled. Throughout the review process (title, abstract and full-text screening stages) the research team met weekly and any uncertainties on article inclusion/exclusion were discussed until consensus was reached.

**Table 1:** Inclusion/exclusion criteria

Inclusion	Exclusion
Population under primary-school age (< 5–7 years depending on country), and over 2 years	Population attending full-time schooling
Outcome is solid food or drink not designed for babies/weaning	Outcome is food/drink designed for babies/weaning stage
Free-living population without a clinical condition	Children with a clinical condition e.g. children with ADHD. Not including obesity
The outcome (dependent) variable is an assessment of food/drink/energy intake	Dietary intake is not an outcome (dependent) variable
Studies in developed countries (as defined by United Nations 2019)	Studies conducted developing countries (as defined by United Nations 2019)
Studies conducted 2000-present	Studies conducted before 2000
Articles published in English	Articles written in a language other than English

### Synthesising the data

The included full text articles underwent data extraction into a standardised table which collected the following information: Year of study and publication, Country of study, Aims/purpose, Study design, Study population (sample size, type e.g. children, parents, nursery staff, age), Intervention (if applicable), Comparison group (if applicable), Independent variable(s), Outcome variable(s), Outcome measurement method, Key findings. As data were extracted the researchers began organising the data into their ecological level of study e.g. individual (child) level, parent level, household level (social or physical environment), childcare centre level or wider determinants (e.g. advertising). Some studies included factors at more than one ecological level in which case they could be included in more than one analysis document, the ecological levels which most commonly overlapped were those at the child and the parental levels. Once studies were organised by ecological level the focus of the studies were clustered. For example, within the child level, all studies which included child food neophobia as an influence on food intakes were identified. At this point the study designs and populations included were summarized in order to provide information on the number and types of studies which address each type of influence on children’s dietary intakes. A summary table of all the included articles is included in Additional file 3. As the aim of a scoping review is not to provide an in-depth synthesis of the results of the papers reviewed but rather to examine the breadth and extent of the evidence available on a topic [15, 16], only key findings (i.e. results from final, fully adjusted models) were included in the review and are summarised in the results section.

## Results

**Fig. 1:**
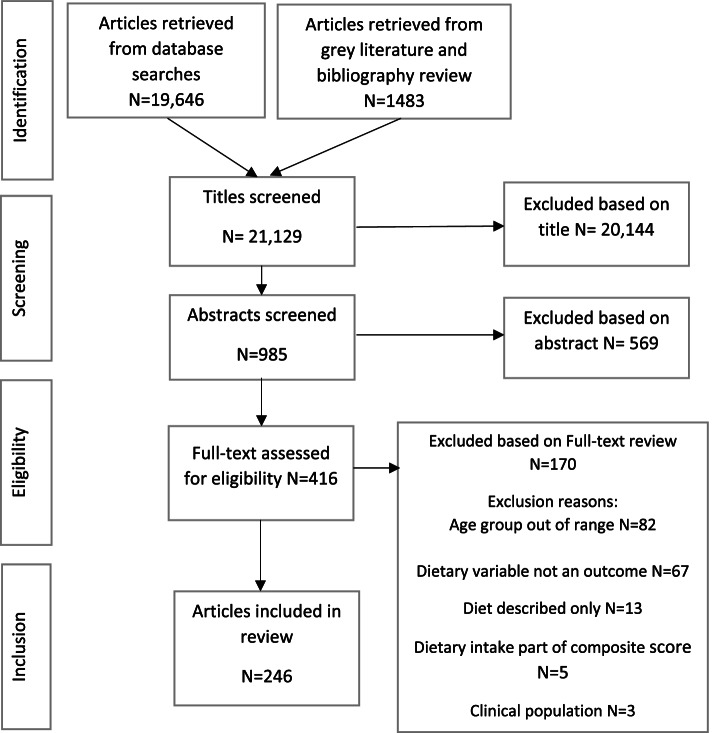
Flow diagram showing the screening process to identify articles for review

The numbers of articles identified and excluded at each process described in the methods is depicted in the flow diagram in Fig. 1. In total 21,129 articles were subject to title review, following which 985 article abstracts were screened. After exclusion of 569 articles based on information in their abstracts 416 full-text articles were screened and a final total of 246 articles were included in the review. The main reasons for exclusion following full-text review was that the participant’s ages fell out of range, or an assessment of dietary intake was not considered an outcome in analyses.

**Fig. 2:**
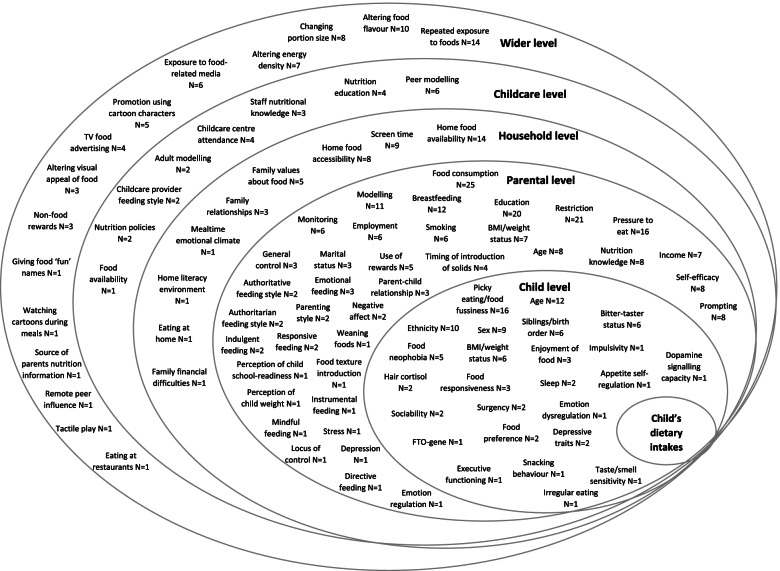
Ecological model showing all factors assessed in relation to children’s dietary intakes in the articles reviewed, and the number of articles which assessed each factor

In total we reviewed 246 articles which assessed the influence of 94 factors on children’s dietary intakes (see Fig. 2). A summary of the 246 articles included in the review is included in Additional file 3. The largest proportion of research was completed in the USA (*n* = 120 49%), followed by the UK (*n* = 39 16%), Australia (*n* = 23 9%) and Netherlands (*n* = 11 5%). Most of the studies were cross-sectional analyses (*n* = 109 44%), followed by experiments (*n* = 50 20%), interventions (*n* = 40 16%), longitudinal analyses (*n* = 36 15%), and observational studies (*n* = 11 5%). A summary of the influences identified are organised by ecological level and described below, the superscript reference numbers refer to each article described in the results section and its position in the summary table in Additional file 3.

### Child level

In total 73 articles reported findings at the child level of which *n* = 39 (53%) were ‘multi-level’ and considered factors at the parent, home and/or wider levels as well. Two thirds of articles were of cross-sectional design (*n* = 46 63%) and the remainder either longitudinal (*n* = 16 22%), experimental (*n* = 9 13%) or interventions (*n* = 1 2%).

#### Child demographics and other characteristics

There were 36 included articles which reported associations between child demographics or other characteristics (e.g. BMI) and diet. Twelve articles included findings related to child age, and the results were largely consistent in that as children’s age increased diet quality tended to decrease ^(4, 7, 31, 54, 60, 82, 94, 120, 126, 150, 189, 244)^.

Nine articles reported associations between child sex and diet, although the findings were mixed. Being male was found to be associated with a less healthy diet ^(128, 169, 170, 232)^ and more eating in the absence of hunger ^(7, 95)^, 2 studies reported a moderation effect ^(68, 95, 232)^. Girls were found to consume more vegetables ^(39, 141)^, or more unhealthy snacks when parents restricted snacks ^(68)^.

Findings related to child’s ethnicity were reported in ten articles and were also conflicting ^(26, 54, 105, 134, 169, 170, 171, 174, 236, 244)^. Most studies compared diets of ‘white’ children with other ethnicities i.e. Asian, Hispanic, African-American. There was no clear direction of the findings and the way in which participants are grouped according to ethnicity is inconsistent across studies.

There were six articles which reported findings in relation to child’s siblings or birth order. The findings were fairly consistent with *n* = 5 studies reporting that children with more, or older, siblings had less healthy diets ^(72, 135, 144, 169, 170)^. In children with no siblings *n* = 1 study reported lower diet diversity and adequacy ^(10)^.

Child BMI/weight status was assessed as a predictor of child diet quality in six studies. Again, the findings were mixed. Being underweight was associated with lower diet diversity and adequacy ^(10)^ and having a higher BMI was associated with a greater food intake when larger portions were offered ^(208)^ and with greater consumption of sugar-sweetened beverages ^(35)^, or energy ^(74)^, compared with healthy-weight counterparts. In contrast, two studies reported null findings, with no associations between child BMI/weight status and intakes of sugar-sweetened beverages ^(31)^ or eating in the absence of hunger ^(7)^. Two articles assessed the relationship between children’s sleep and diet quality ^(113, 155)^. One study found that variation in weekend versus weekday sleep was associated with indicators of poorer diet quality ^(113)^ and the other study reported shorter sleep duration was positively associated with eating in the absence of hunger ^(155)^.

#### Child biological characteristics

There were 10 articles which assessed biological traits of the child in relation to food intakes. More than half of these (*n* = 6) focused on genotypes or polymorphisms related to the ability to taste bitter compounds ^(12, 34, 103, 119, 168, 183)^. Of these studies, four consistently found that children who were genetically predisposed to taste bitter compounds more strongly consumed more foods high in sugar ^(34, 103, 119, 183)^. One study reported that ‘non-tasters’ were more likely to consume bitter vegetables than tasters ^(12)^ however other studies did not find differences in vegetable consumption between groups ^(103, 119)^ and one intervention found that increased exposure to turnip was associated with an increase in consumption, regardless of taste geno- or phenotypes ^(168)^.

Two studies assessed child hair cortisol concentrations, as a marker for stress, finding that higher cortisol concentrations were associated with a less healthy diet. ^(131, 220)^

One study each focused on assessing variations in, dopamine signalling capacity ^(206)^, and the FTO-gene ^(233)^ and reported positive associations with children’s sugar intake, and sweet-biscuit intake, respectively.

#### Child non-food related psychological characteristics

Non-food related psychological characteristics were investigated in 8 studies and included depressive and anxious traits ^(90, 225)^, sociability ^(101, 222)^, surgency ^(118, 225)^, impulsivity ^(15)^, emotion dysregulation ^(106)^ and executive functioning ^(191)^. The two articles which assessed depressive/anxious traits were conflicting, as were the results reported in the studies assessing sociability, and surgency, with vegetable consumption. Finally, studies assessing impulsivity or executive functioning showed no significant association with children’s diet. However, the study assessing emotion dysregulation ^(106)^ showed that higher child emotion dysregulation was associated with more obesogenic food consumption, but only when their caregiver’s emotion dysregulation was also high.

#### Child eating behaviours

A total of 29 articles assessed children’s eating behaviour and diet. The majority of these assessed ‘picky eating/food fussiness’ (*n* = 16, 55%) ^(18, 22, 28, 33, 56, 75, 101, 102, 105, 116, 171, 189, 198, 215, 218, 223)^. Food neophobia was assessed in five articles ^(38, 39, 115, 157, 232)^ and others considered food responsiveness ^(18, 90, 208)^, food preference ^(90, 149)^, perceived taste/smell sensitivity ^(43)^, enjoyment of food ^(39, 75, 222)^, over- and irregular eating ^(56)^, snacking behaviours ^(72)^ and appetitive self-regulation ^(83)^. All articles reported consistent findings that children who were perceived to be more picky/fussy eaters had poorer diet quality ^(18, 22, 28, 33, 56, 75, 101, 102, 105, 171, 189, 196, 218, 223)^. Although conceptually separate, similar relationships were reported between child food neophobia and diet, with those who were more neophobic consuming fewer fruits and vegetables ^(38, 39, 115, 232)^ or being less likely to try a novel vegetable ^(157)^.

Consistently, articles reported that food responsiveness had a positive relationship with diet quality and/or novel food acceptance ^(18, 90, 208)^. Furthermore, children who had higher ratings of their liking of fruits and vegetables ^(90, 149)^, and those who enjoyed their food more ^(39, 222)^, tended to consume more fruits and vegetables. However, one study showed that enjoyment of food was associated with higher energy intakes, but moderation analyses showed this only to be significant in children with a high BMI ^(75)^.

### Parental level

In total, 118 papers examined factors at the parental level and their impact on children’s diet. Of these, 39% (*n* = 46) were multi-level studies, considering the relative influence of factors at other ecological levels as well. Most were cross-sectional by design (61% *n* = 72), with 14% (*n* = 17) longitudinal, 12% (*n* = 14) interventions, 7% (*n* = 8) experimental and 5% (*n* = 6) observational.

#### Parent demographics

A total of 28 studies reported findings focused on parental demographic characteristics and children’s diet. Parental education was the most commonly reported in *n* = 20 studies with entirely consistent results showing a positive relationship between parental education and children’s diet quality ^(7, 10, 72, 89, 108, 128, 130, 137, 149, 154, 164, 169, 170, 171, 177, 179, 188, 200, 221, 237)^. Other socioeconomic factors studied included income, employment, marital status and housing tenure. Household income was assessed in *n* = 7 studies. Results were consistent with studies reporting that lower incomes were associated with poorer diet quality ^(72, 81, 130, 169, 171,177, 200)^. Parental employment was a focus of 6 studies with conflicting results. Maternal employment has been shown to be associated with poorer patterns of diet in children ^(169, 185)^. On the other hand, having parents working has been associated with greater fruit and vegetable intakes in children ^(177)^. Furthermore, children of non-working mothers have been shown to have lower ^(179)^ and higher ^(171)^ intakes of junk foods. Marital status was reported in 3 studies, with consistent results: children with mothers who were married/cohabiting tended to have better diets than those who were single ^(171, 177, 226)^.

Parental age was reported in *n* = 8 studies, which were consistent in their overall findings. Some studies showed that younger parents tended to have children who had more unhealthy dietary patterns ^(128,177, 226)^, or consumed more SSB ^(130)^. Furthermore some studies reported that older parents tended to have children who had a better quality diet ^(137, 169, 171, 177)^. Parental (rather than child) ethnicity was a focus of 5 studies ^(105, 135, 164, 169, 171)^. The results were mixed with no clear directions of an association. The relationship between maternal smoking status (both current status and during pregnancy) and children’s diet was assessed in 6 studies. The results were consistent with smoking status being inversely associated with aspects of children’s diet quality ^(72, 128, 135, 169, 170, 226)^.

Parental BMI and children’s diet quality was the focus of 7 studies with consistent findings. Lower maternal BMI has been associated with better overall diet quality in children ^(72)^. In contrast, higher parental BMI has been associated with increased sugar ^(60, 143)^, junk food ^(64, 185, 226**)**^ and snack intakes ^(89)^ in children.

#### Parental food consumption

In total, 25 studies focused on the relationship between parents’ dietary intakes and children’s dietary intakes. Whether the studies assessed parent and child’s whole dietary patterns ^(72, 94, 130,224)^, fruit and vegetable intakes ^(39, 43, 44, 84, 102, 105, 117, 136, 149, 221, 222, 232, 241)^ snack intakes ^(149, 237)^, sugar-sweetened beverage intakes ^(36, 81, 221, 237)^, or other dietary elements ^(47, 55, 62, 66, 129, 237)^, every study reported a positive relationship between the dietary intakes of parents and that of their young children.

#### Parent psychological characteristics

Psychological characteristics of the parents and their relationship to child dietary intakes were reported in *n* = 17 studies. The most common psychological characteristic studied was parental self-efficacy (a person’s belief in their ability to carry out a behaviour), which was reported in 8 studies. There was considerable variation in the domains of self-efficacy assessed and no clear direction of effects could be concluded, with 4 studies reporting a positive relationship with children’s diet quality ^(85, 114, 197, 243)^ and 4 reporting no association ^(36, 37, 97, 136)^. Three studies examined parental perceived general control ^(36, 114, 150)^, of which 2 reported a positive relationship with children’s quality of diet ^(114, 150)^. A further three studies ^(109, 151, 234)^ demonstrated that a poorer parent-child relationship was consistently, negatively associated with children’s diet quality. Two studies showed that greater negative affectivity in parents was associated with poorer diet quality in children ^(225, 245).^ Two studies also assessed parenting style ^(151, 243)^ showing that maternal warmth was associated with greater fruit and vegetable consumption in children, whereas parental hostility was associated with greater snack intake. Negative emotion regulation ^(24)^, depressive symptoms ^(230)^, parental locus of control ^(245)^, and stress ^(234)^ were all associated with aspects of a poorer diet in children. In contrast, parent perception of child weight status ^(136)^ and their child’s school readiness ^(214)^ were both associated with greater fruit and vegetable intakes in children. The limited number of studies focusing on these individual characteristics means consistency of findings cannot be evaluated.

#### Early feeding practices

Early feeding practices refers to the way in which parents fed their children when they were infants. This has been reported in relation to children’s dietary intakes in the preschool years in 14 studies included in this review. Most studies (*n* = 12) reported the associations between breastfeeding duration and later child diet, with consistent results ^(10, 23, 37, 39, 50, 76, 128, 135, 137, 158, 161, 215)^. Mothers who breastfed their child for longer had children with better quality diets. Other early feeding practices studied included timing of introduction of solids ^(37, 76, 158, 215)^, number and type of foods tried during introduction of solids ^(146)^ and food texture introduction ^(171)^ although the results between these factors and children’s diet quality were inconsistent, with no clear direction of effect.

#### Feeding practices

Feeding practices refer to the types of strategies used by parents to manage their children’s dietary intakes. The relationship between parental feeding practices and children’s diets has been extensively studied with *n* = 44 articles identified in our review. Feeding practices studied included pressure to eat, modelling food intake, restriction of food, prompting/encouragement, monitoring, use of rewards and use of food for emotion regulation. Largely consistent findings have been reported in studies assessing pressure to eat (*n* = 16 studies ^(8, 58, 70, 80, 87, 95, 101, 111, 112, 116, 141, 151, 222, 223, 232, 245)^), modelling (*n* = 11 studies ^(18, 37, 84, 87, 100, 101, 116, 141, 187, 205, 220)^) and prompting/encouragement of food intakes (*n* = 8 studies ^(18, 59, 79, 116, 143, 149, 205, 222)^). Pressure to eat has largely been associated with aspects of poorer quality diets in children, although 2 studies reported null associations ^(111, 223)^. Parental modelling (eating the same food as the child at the same time) and prompting/encouragement have largely been associated with aspects of better quality diets in children. Parental use of restriction of foods in relation to children’s dietary intakes was the most frequently assessed feeding practice, reported in 20 studies ^(16, 20, 21, 42, 58, 68, 69, 78, 87, 90, 95, 111, 115, 160, 181, 187, 205, 223, 232, 245)^ but the findings were equivocal. However, those studies which separated out types of restriction into categories of overt (obvious to the child) and covert (less obvious to the child) (*n* = 5) yielded consistent findings, with covert restriction being associated with better quality diets compared to overt restriction ^(20, 21, 58, 115, 160)^. Parental monitoring of food intake was reported in 6 studies with no consistent effects reported ^(15, 58, 95, 112,149, 205)^. Use of a reward (either food or non-food based) was reported in *n* = 5 studies, which found that use of food-based rewards was associated with aspects of poorer diet quality ^(122, 139, 221)^ whereas non-food rewards e.g. praise, were associated with greater vegetable consumption ^(100, 116, 221)^. Three studies reported consistent inverse associations between use of food for emotion regulation and children’s dietary quality^(17, 122, 139)^. Finally *n* = 12 studies analysed moderating or mediating effects of some child or parental characteristics on the relationship between parental feeding practices and children’s dietary quality ^(15, 18, 42, 68, 70, 90, 95, 116, 118, 143, 181,232)^. For example, parental pressure to eat was only found to be associated with eating in the absence of hunger in boys ^(95)^, whereas restriction of snacks was associated with increased snack intake only in girls ^(68)^. Furthermore, maternal prompts and modelling of novel food was associated with a higher likelihood of novel food acceptance in higher food responsive children ^(18)^, in another study novel food acceptance was higher following maternal prompts in children whose mothers had obesity ^(143)^.

#### Feeding style

Parental feeding styles refer to the way in which parents interact with children around food within the framework of general parenting styles ^(99)^. The relationship between parental feeding styles and children’s dietary intakes were the focus of 9 studies in the review. Parental feeding styles were assessed in a number of different ways, with no one measure being more frequently used than another, so findings with children’s dietary intakes were mixed. Two studies indicated that authoritative feeding style was positively associated with fruit and vegetable intakes ^(6, 182)^, whereas authoritarian ^(6, 182)^, indulgent or uninvolved ^(99, 181)^ feeding styles were inversely associated with aspects of children’s dietary quality in the studies which included these assessments. Parental responsiveness and demandingness showed null results with children’s diet quality in the two studies which assessed these ^(20, 140)^. Finally, non-directive feeding ^(174)^, mindful feeding ^(60)^, and encouraging, feeding styles ^(108)^ were all associated with better quality diets, whereas instrumental feeding style ^(108)^ was associated with poorer diet quality.

#### Parent level interventions

We identified 15 intervention studies in which parents were the target for improving children’s dietary intakes ^(11, 49, 57, 93, 125, 145, 166, 172, 176, 194, 216, 217, 227, 238, 242)^. Exact mechanisms targeted in the intervention studies varied, although there were some general similarities, with the most common intervention targets being parents’ nutrition education/knowledge (*n* = 8 ^(11, 49, 57, 125, 166, 172, 194, 238)^) and/or parental feeding practices (*n* = 6 ^(49, 93, 145, 176, 216, 227)^). The delivery of the interventions also greatly varied with some using printed newsletters ^(93, 194, 216, 242)^, text messages ^(166)^, phone calls ^(216, 242)^ websites ^(125, 166)^, mobile phone apps ^(172)^, CD’s ^(57, 172)^ and/or face-to-face interactions ^(11, 49, 93, 145, 166, 217, 238)^. Effects on children’s dietary quality were inconsistent due to variation in delivery, intensity, length to follow-up and outcome assessed. However there was some evidence that increasing parental nutritional knowledge ^(49, 57)^ and/or parental modelling of fruit and vegetable consumption ^(49, 93)^ was associated with improvements in children’s dietary outcomes.

### Household level

There were 38 studies which reported associations between factors at the household level and children’s quality of diet. Of these 65% (*n* = 24) were multi-level studies which assessed the relative influence of factors at the parent and/or child level as well. The majority of studies were cross-sectional analyses (*n* = 28; 74%), with the remainder being longitudinal (*n* = 7; 18%) or intervention studies (*n* = 3; 7%).

#### Family characteristics

The relationship between various family characteristics and children’s dietary intakes were assessed in 10 studies, although there were few similarities between the studies. Three studies considered factors relating to family relationships i.e. family functioning ^(164)^, family cohesion ^(148)^ and/or household chaos ^(7, 148)^. Higher family functioning and cohesion were associated with aspects of better diet quality in children ^(148, 164)^, whereas household chaos showed mixed findings related to child diet in the 2 studies which reported it ^(7, 148)^. Five studies focused on the perceived importance families gave to food e.g. family food involvement ^(152)^, having policies about eating as a family ^(179)^, or frequency of family meals ^(47, 114, 235)^, all of which were associated with aspects of better diet quality in children. Finally, only one study each considered the influence of mealtime emotional climate ^(199)^, and families’ perceived financial difficulty ^(207)^. A positive emotional climate was associated with higher healthy food consumption ^(199)^, whereas more financial difficulty was associated with poorer diet quality ^(207)^.

#### Food availability and accessibility

The most frequently studied factors at the household level was home food availability (*n* = 13 studies) and accessibility (*n* = 7 studies). Home food availability refers to whether a food group or item(s) are present within the child’s home (e.g. there is fruit at home), whereas food accessibility refers to whether a food group or item(s) are accessible by the child (e.g. there is a fruit bowl within the child’s reach). Associations reported between food availability and children’s quality of diet were largely consistent with 11 of the 13 studies reporting that higher availability of a food group e.g. fruit and vegetables, and/or SSB, was associated with higher consumption of that food group ^(19, 29, 66, 84, 129, 134, 140, 149, 174, 204, 212, 241)^. One study reported non-significant relationship between food availability and children’s overall quality of diet ^(37)^. Similarly, of the 7 studies which reported relationships between food accessibility and children’s dietary intakes, 5 found consistent relationships with either greater accessibility related to greater intakes of that food ^(85, 204, 241)^, or that restricting access was related to lower consumption ^(179, 212)^. One study reported no significant effect ^(19)^, and one study found that accessibility moderated a relationship between parental use of restriction and children’s eating in the absence of hunger ^(42)^.

#### Home environment

A total of 11 studies reported associations between aspects of the physical home environment and children’s dietary intakes. The majority (71%; *n* = 9) focused on child’s use of screen time or television viewing either as a total time spent using a screen ^(10, 114, 135, 147, 149, 153, 237)^ or specifically during mealtimes ^(175, 235)^. All 9 studies reported a consistent relationship that higher frequencies of screen time was negatively associated with aspects of children’s diet quality. Other aspects of the home environment considered were the home literacy environment ^(184)^ which was positively associated with children’s fruit and vegetable intakes, and the calories consumed from snacks provided in the home versus out-of-home ^(110)^ indicating that out-of-home snacks were more calorie dense.

#### Household level interventions

Three intervention studies specifically focused on aspects of the home environment to change children’s’ dietary intakes. Two interventions involved home visits ^(93, 156)^, whereas in the other parents received the intervention over the phone ^(240)^. Two of the interventions focused on fruit and vegetable availability or accessibility/provision and the other intervention allowed parents to choose the focus (either limiting screen-time, increasing family meals, making time for physical activity or improving the bedtime routine). Both interventions which improved home fruit and vegetable availability or provision reported some increases in children’s intakes of these, relative to the control group ^(93, 156)^.

### Childcare level

Of 24 studies which examined factors at the childcare level related to quality of children’s diets, 7 studies focused on modelling (either peer or adult) occurring within childcare settings, 8 evaluated interventions which took place in childcare settings, and the remaining 9 examined factors in the broader childcare environment (e.g., number of days in childcare, care provider’s feeding practices, education). Of the 24 studies, 4 were ‘multi-level’ and considered the relative influence of factors at child, parent, household or wider determinant levels. At this level 33% (*n* = 8) were cross-sectional analyses, 33% (n = 8) were interventions, 17% (*n* = 4) were experimental, 13% (*n* = 3) were observational and 4% (*n* = 1) was longitudinal.

#### Modelling

Modelling studies examined the effect of peer models (*n* = 5) and adult models (*n* = 2) on children’s dietary quality. Five studies reported that children model their peers’ eating behaviour ^(86, 98, 189, 213, 229)^. One study reported that female peer models influenced eating behaviour, but male peer models did not ^(98)^. There was variation in the longevity of peer modelling effects ^(98, 229)^. Two studies found a positive effect of adult modelling on children’s dietary intakes ^**(**1, 229**)**^.

#### Childcare environment

A variety of factors within the childcare environment were studied. One of the nine studies reported that preschool nutrition education for children was associated with better dietary quality ^(^and there was a moderation effect by maternal educational level ^(117)^. Childcare staff’s nutritional knowledge was assessed in one study that showed a positive relationship with children’s dietary diversity and quality ^(14)^. Four studies reported on the association between attendance at childcare centres and diet quality, suggesting that greater attendance was associated with better dietary quality ^(9, 82, 92, 159)^. Inconsistent findings were reported about the effect of childcare staff’s feeding style on children’s dietary quality ^(91, 107)^. Three studies reported on the availability and accessibility of food, or food-based policies within the childcare setting reporting a positive association with children’s diets ^(9, 14, 189)^.

#### Interventions in childcare settings

Eight studies utilised an intervention design in the childcare setting. Three studies manipulated the food presented to children using exposure, modelling and/or reward. Of these, one study found that modelling combined with reward was effective at increasing fruit and vegetable consumption ^(104)^. The effect of exposure on vegetable intake had mixed findings in two studies ^(173, 246)^. Interventions that provided children with nutrition focused education (*n* = 3) reported mixed findings of the effect on F&V consumption ^(88, 163, 237)^. Two studies focused on providing childcare staff with nutrition education ^(13, 238)^, however these were part of an intervention package which involved teaching children and parents as well and so the relative influence of training the childcare staff could not be determined.

### Wider level

The wider determinants of children’s dietary quality have been separated into 2 categories. External influences are those beyond the child, parent, household and childcare ecological levels e.g. cartoon characters on packaging or in adverts, which could potentially influence a child in a variety of settings. The second category is ‘manipulation of food served’, these studies assessed a broad concept of how changing the way that a food is served may affect child’s intake, which is also an influence which has been studied independent of ecological level and could potentially occur in a variety of levels (for example, manipulating portion sizes in household or childcare settings). There were 55 studies which considered wider level influences on children’s dietary intakes, *n* = 21 which were classed as external influences and *n* = 34 which manipulated food served to children. At this level most studies were experimental (*n* = 38, 69%), with the remainder being interventions (*n* = 11, 20%), longitudinal (*n* = 3, 5%) or cross-sectional (*n* = 3 5%).

#### External influences

Exposure was the most commonly assessed external influence. The effect of increasing exposure to foods via non-food related methods was assessed in *n* = 6 studies. These included promoting fruit and/or vegetables via: a maths game mobile phone app ^(65)^, story/picture book ^(30, 180)^, motivational theatre (puppet show) ^(177)^, placemats ^(192)^, or sensory play ^(45)^. All of these studies reported positive results with increases in children’s fruit and/or vegetable consumption. In total 5 studies assessed the effect of using cartoon characters to promote foods on children’s intake of that food ^(46, 121, 127, 186, 193)^. The results were largely consistent showing that using cartoon characters resulted in increased consumption of that food in pre/post experiments, although one study reported no effect ^(186)^. The effect of children’s exposure to food advertisements on television and their food intakes was reported in 4 studies ^(48, 61, 62, 63)^. These all focused on advertisements for less healthy foods and reported positive associations with less healthy food consumption ^(48, 62, 63)^ or eating in the absence of hunger ^(61)^.

The effect of offering a non-food reward to consume healthy food was assessed in 3 studies and showed a consistent positive effect on children’s food intake ^(40, 67, 190)^. Finally, one study each reported positive associations between children’s dietary intakes and giving healthy food ‘fun’ names ^(162)^, and tactile play enjoyment ^(44)^. There was also a positive relationship reported between children’s beverage intake and that of their peers, even when the peer was remote ^(123)^. Dietary intakes indicative of poorer quality diets were reported in children who ate more in restaurants versus home ^(159)^, watched a cartoon whilst eating ^(77)^, and whose mother reported the child’s grandmother as their main source of feeding/nutrition information ^(126)^.

#### Manipulation of food

The studies which tested the effect of manipulating foods on children’s food intakes could be grouped into 5 sub-categories: 1) alteration of portion size, 2) increasing exposure using food-based methods, 3) alteration of a food flavour by pairing it with another food e.g. serving vegetables with a dip, 4) alteration of energy density, or 5) alteration of visual appearance.

The effects of portion size on children’s intake was the focus for *n* = 8 studies ^(124, 138, 178, 201, 203, 208, 209, 211)^ of which the results were consistent: when larger portions were offered to children more of the food (or increased kcal) tended to be consumed. There was some suggestion that child BMI may moderate this association ^(201, 208)^. The effect of repeated exposure to foods (using the physical food itself) on children’s food intakes was reported in *n* = 14 studies, which also showed consistent effects. Children tended to consume more of the food following repeated exposure to it ^(3, 5, 25, 51, 52, 53, 67, 71, 73, 96, 165, 168, 195, 231)^. The effect of altering the flavour of foods on children’s food intake was reported in *n* = 10 studies, however the results were mixed. Most identified a positive effect on overall intake of the altered food (always a vegetable) ^(5, 25, 32, 52, 96, 198, 202)^ whereas two showed no effect ^(2, 41)^ and one only found an effect in bitter-sensitive children ^(124)^. Altering the energy density of foods was assessed in *n* = 7 studies with mixed results. Four of the 7 studies reported no significant effect of altering energy density on children’s food intakes ^(51, 96, 124, 138)^, whereas 3 studies found a beneficial effect ^(132, 133, 210)^. Finally altering the visual appeal of food was assessed in 3 studies. There was no effect of altering the visual appeal of foods on children’s food intakes ^(26, 27, 41)^.

## Discussion

In this article we have collated and summarised the breadth of evidence generated over the last 20 years assessing key influences on dietary intakes of preschool children in developed countries. The articles reviewed had identified multiple factors at many ecological levels, and thus highlights the complexity of the system that shapes the establishment of dietary habits in preschoolers. Although a formal assessment of the quality of the evidence is beyond the scope of a systematic scoping review, it is of note that study designs and outcome assessments varied widely. The majority of the evidence comes from cross-sectional surveys, followed by experiments, longitudinal surveys and interventions. Despite the wealth of evidence from cross-sectional studies, the direction of the association is inferred, which cannot be determined from this type of study design. In fact many of the associations could be causal in the opposite direction than suggested, exist in feedback loops, or not be causally related at all. The application of systems modelling analyses e.g. agent-based modelling, would allow exploration of the dynamic relationships between these factors and is an important next step in the evaluation of children’s dietary intakes. The outcome assessed also varied greatly. Most studies focused on dietary intakes which were indicative of a more or less healthy diet i.e. fruit and vegetable, and/or energy-dense nutrient-poor food intakes, with fewer studies assessing quality of whole diet. Furthermore, most used subjective, parent-reported, methods of dietary assessment, with the exception of experimental studies which tended to use objective measures e.g. weighed portions. A wealth of research has assessed the validity (or relative validity) of various parent-report dietary assessment methods and most conclude that subjective assessments are adequate for ranking populations according to diet quality/food group consumption [20]. Therefore, from a systems thinking perspective, the evidence can be collated to reflect influences which are generally beneficial or detrimental to children’s quality of diet. However, were the focus on more precise outcomes e.g. energy intake, capacity for collating the findings of the literature is challenged by both the heterogeneity of outcomes assessed and the methods of their assessment across the evidence base.

Factors affecting child dietary quality at the child level were commonly studied, including those of biological, demographic and psychological origin. There were few consistent findings within the factors at this level. However, the associations between food pickiness/neophobia on children’s dietary intakes were overwhelmingly the most common focus and these associations were firmly consistent, showing that picky or neophobic eaters tend to have poorer diet quality. Whilst there were some studies which considered the moderating effects of child eating behaviours in the relationship between other factors e.g. parental feeding practices and children’s diet quality, the evidence available on these interactions is scarce. From what is available it seems that influences on children’s diet quality may work differently according to children’s levels of food fussiness or food responsiveness [21, 22]. Differences in the way in which parents feed their child according to child traits such as food fussiness or temperament, have been the focus for a number of studies [23–25], however most do not go on to explore those effects on dietary outcomes and therefore could not be considered in this review. The role that differences in children’s food-related psychological traits play in interactions with parental and environmental influences would be important considerations when simulating the system of influences on children’s diet quality, in order to better understand what works, for whom and under what circumstances.

Of the 246 included articles the majority (*n* = 118) included factors at the parental level. This is understandable as parents tend to be responsible for providing food on behalf of their child [26]. Although studies often define influences at the ‘parent’ level, the majority of respondents in these types of study are mothers, although not all studies reported the proportion of mothers in their participant characteristics. Only two studies included in this review specifically focused on the role of fathers so there is a clear gap in the literature [27, 28]. The effects of both caregivers, individually and/or together, within the system of influence could be important to untangle. At the parental level ‘feeding practices’ and ‘demographics’ were the most commonly assessed influences, and factors within these themes also provided the most consistent evidence. Perhaps unsurprisingly, this meant that some interventions at the parental level aimed to target feeding practices. Although more interventions were targeted at increasing nutrition education/knowledge of parents, from our review it is unclear as to whether a lack of nutrition knowledge was actually a determinant of poorer diet quality in children. As highlighted in a recent Cochrane review (2020) [29], interventions aimed at improving fruit and vegetable intakes of under five-year-olds have had varied effects, with poorly sustained results. This suggests that we do not yet know the best target, or delivery, for intervention, or how to tailor interventions effectively for different families. A mapping and simulation of the system would aid in future intervention design. Whilst demographic associations such as educational level and household income are well documented, they serve to address ‘who’ interventions could be targeted to, rather than ‘what’ the target should be, because demographics are deemed less malleable than behaviours such as feeding practices.

At the household level, factors beyond the parents but within the home were assessed, although many of these factors are likely controlled by parents. The largest body of evidence at this level came from the home availability of foods, and use of screen time. Fewer studies focused on the effects of the mealtime environment or other family members within the home. There was limited evidence on how factors in the home environment interacted with factors at the parent and/or child level, and their combined association with children’s dietary intakes. Given that preschool children are still likely to consume most food at home [30] and with members of their family, it will be important for future research to consider the interacting effects of factors within the home, parent and child on children’s diet quality. Studies to date largely consider factors contained within one or two ecological levels, and where multilevel studies have been conducted, analyses tend to only consider the relative influence of multi-level factors, rather than assessing any interacting effects. For instance factors at the child, parental and home level have been considered in some articles, however all variables are considered covariates in multivariate regression models, without consideration of interactions between these variables on the outcome [31, 32].

Whilst childcare settings were often the location to complete research studies assessing influences on children’s quality of diet, it was not often the effects of childcare per se which was the focus. With only 4 of the 24 studies on the influence of childcare settings on children’s diets considering multi-level factors, findings at this level were infrequent and isolated, in comparison to studies at the other ecological levels. Data from the UK, Australia, the USA and Canada suggest that around 60–80% of 3–4 year old children attend a formal childcare setting [33–36], so this is an important, missing piece of the puzzle. The evidence of the influences of childcare settings on children’s dietary intakes was split between the impact of modelling food intake (by peers or adults), and factors within the environment e.g. healthy eating policies, or provision of nutrition education. The relative impact of childcare settings on children’s dietary intakes is difficult to establish given that childcare settings vary widely and as a result there are a number of factors which will determine their nutrition environment, i.e. who regulates the setting, staff nutrition/food knowledge, budget allocated to food provision, etc. [37, 38]. However, characterising childcare settings, formal and informal, and their interactions with factors at the wider and individual levels, to examine their impact on children’s dietary intakes, remains a fundamental gap in the literature.

Most of the evidence grouped at the ‘wider’ level concerned experiments on the effect of the broad concept of manipulating food served to children e.g. altering the presentation of foods, rather than influences within the wider society. Altering food flavours or presentations seems to be an efficacious way of slightly increasing intakes of previously disliked, unknown, or under-consumed foods in small scale experiments. However, the effectiveness of scaling up these approaches is yet to be established.

At a national or international level, large-scale public health policies and initiatives, often aim to target wider societal factors e.g. TV advertising [39], or taxation of less healthy foods [40], however, at the preschool age, evidence for the potential impact of these initiatives is largely lacking. This is partly because it is particularly challenging to evaluate the impact of wider societal level factors on outcomes at an individual level, when the ecological levels are so distal and individual effects are likely mediated or moderated by other elements within the system, such as those at the household and parental levels. The appreciation of this complexity is not achieved by traditional epidemiologic analyses. This is a key consideration for future systems approaches focused on improving children’s diet quality, because there is not a one-size-fits-all solution. Better insight into how to evaluate changes at the wider societal level on impacts at the individual level, will allow researchers to simulate anticipated effects from future public health policies and initiatives.

### Strengths and limitations

The literature was systematically searched and screened following an a priori protocol, and guidelines published by the Joanna Briggs institute [15, 16]. A random sample of 20 abstracts, and all full-text articles were reviewed by two reviewers, independently. There are no definitive guidelines for completing a systematic scoping review and other frameworks have been published, including that by Arksey and O’Malley (2005) [41], which was extended by Levac et al. (2010) [42]. Whilst most of the steps in the Levac et al. framework mirror very closely with those in the guidance from the Joanna Briggs institute, Levac et al. recommend a incorporating consultation with stakeholders as part of the knowledge translation component, which the Joanna Briggs guidance does not. Therefore, it is possible that the knowledge translation of our review could have been strengthened if we had followed the Levac framework. Whilst a scoping review aims to be inclusive, it was necessary to establish exclusion criteria. It is a clear limitation that we excluded studies conducted in middle and low-income countries, however, due to the different drivers of diet quality in these populations, it was beyond the scope for this review. A systematic scoping review of the system influences on diet quality of young children in middle and low-income countries therefore remains an important gap in the literature. Furthermore narrowing the review to include only articles published since 2000 was a decision taken to limit the size of this review and therefore the quantity of evidence for some of the influences may be underestimated. Whilst we excluded studies conducted with children with clinical conditions, due to their unique effect of dietary intakes, we did not exclude based on weight status even though obesity is considered a disease. This is because most studies include populations with mixed weight status and do not stratify analyses based on weight status. Thus, to have excluded studies based on populations which included children with obesity would have limited the review greatly. However it should be noted that the findings in this review include studies of children with mixed weight status even though some of the drivers of dietary intakes may differ between these populations. Finally, as stated in the guidelines for conducting systematic scoping reviews, it is not the aim of a scoping review to assess the quality of the evidence available [15, 16].

## Conclusions

Most of the evidence to date on influences on preschool children’s dietary intakes exists at the individual and parental level. It is important for us to understand this as a system in itself to estimate how wider system changes could translate into individual behaviour changes. There has been substantial debate between researchers and policy makers about whether influences on food choices and subsequent interventions should be targeted at the ‘environment’ or ‘individual’, however this debate fails to recognise the whole system at play. The effects of more distal public health initiatives are likely mediated and moderated by factors at the individual parent/child level. Individual children do not exist within silos and complex interactions between ecological levels mean that interventions may have different effects for different children in different circumstances. Going forwards, methods such as agent-based modelling will allow us to simulate these complex adaptive systems so we can evaluate these phenomena more accurately. It is clear that the last 20 years has generated a wealth of research on influences on preschool children’s diet quality, but in order to generate evidence for a systems thinking approach, a greater understanding of the interactions between factors, across ecological levels, and over time, is warranted.

## Supplementary Information

**Additional file 1.**

**Additional file 2.**

**Additional file 3.**

## Data Availability

Data charting table is available as Additional File 3.

## References

[1] Tandon PS, Tovar A, Jayasuriya AT, Welker E, Schober DJ, Copeland K, Dev DA, Murriel AL, Amso D, Ward DS (2016). The relationship between physical activity and diet and young children’s cognitive development: A systematic review. Prev Med Rep.

[2] Nasreddine L, Shatila H, Itani L, Hwalla N, Jomaa L, Naja F (2019). A traditional dietary pattern is associated with lower odds of overweight and obesity among preschool children in Lebanon: a cross-sectional study. Eur J Nutr.

[3] Scharf RJ, DeBoer MD (2016). Sugar-sweetened beverages and Children's health. Annu Rev Public Health.

[4] Mikkilä V, Räsänen L, Raitakari OT, Pietinen P, Viikari J (2005). Consistent dietary patterns identified from childhood to adulthood: the cardiovascular risk in young Finns study. Br J Nutr.

[5] Scientific Advisory Committee on Nutrition (SACN). Report: Drastic Action on Sugar Consumption Recommended. 2015 Available from URL: https://www.bda.uk.com/news/view?id=75

[6] Welker EB, Jacquier EF, Catellier DJ, Anater AS, Story MT (2018). Room for Improvement Remains in Food Consumption Patterns of Young Children Aged 2–4 Years. J Nutr.

[7] Jarman M, Vashi N, Angus A, Bell RC, Giesbrecht GF, APrON study team (2020). Development of a diet quality index to assess adherence to Canadian dietary recommendations in 3-year-old children. Public Health Nutr.

[8] Lycett K, Miller A, Knox A, Dunn S, Kerr JA, Sung V, Wake M (2017). ‘Nudge’ interventions for improving children's dietary behaviors in the home: A systematic review. Obesity Med.

[9] Holley CE, Farrow C, Haycraft E (2017). A Systematic Review of Methods for Increasing Vegetable Consumption in Early Childhood. Curr Nutr Rep.

[10] Nekitsing C, Blundell-Birtill P, Cockroft JE, Hetherington MM (2018). Systematic review and meta-analysis of strategies to increase vegetable consumption in preschool children aged 2-5 years. Appetite.

[11] Public Health England (2019). Whole Systems Approach to Obestiy: a guide to support local approaches to promoting healthy weight.

[12] Lee BY, Bartsch SM, Mui Y, Haidari LA, Spiker ML, Gittelsohn J (2017). A systems approach to obesity. Nutr Rev.

[13] Bagnall AM, Radley D, Jones R, Gately P, Nobles J, Van Dijk M, Blackshaw J, Montel S, Sahota P (2019). Whole systems approaches to obesity and other complex public health challenges: a systematic review. BMC Public Health.

[14] Powell KE, Kibbe DL, Ferencik R, Soderquist C, Phillips MA, Vall EA, Minyard KJ (2017). Systems Thinking and Simulation Modeling to Inform Childhood Obesity Policy and Practice. Public Health Rep.

[15] Peters MD, Godfrey CM, Khalil H, McInerney P, Parker D, Soares CB (2015). Guidance for conducting systematic scoping reviews. Int J Evid Based Healthc.

[16] Munn Z, Peters MDJ, Stern S, Tufanaru C, McAuthur A, Aromataris E (2018). Systematic review or scoping review? Guidance for authors when choosing between a systematic or scoping review approach. BMC Med Res Methodol.

[17] Kepper MM, Myers CA, Denstel KD, Hunter RF, Guan W, Broyles ST (2019). The neighborhood social environment and physical activity: a systematic scoping review. Int J Behav Nutr Phys Act.

[18] Davison KK, Birch LL (2001). Childhood overweight: a contextual model and recommendations for future research. Obes Rev.

[19] Tricco AC, Lillie E, Zarin W, O'Brien KK, Colquhoun H, Levac D (2018). PRISMA extension for scoping reviews (PRISMAScR): checklist and explanation. Ann Intern Med.

[20] Serdula MK, Alexander MP, Scanlon KS, Bowman BA (2001). What are preschool children eating? A review of dietary assessment. Annu Rev Nutr.

[21] Jordan AA, Appugliese DP, Miller AL, Lumeng JC, Rosenblum KL, Pesch MH (2020). Maternal prompting types and child vegetable intake: exploring the moderating role of picky eating. Appetite.

[22] Blissett J, Bennett C, Fogel A, Harris G, Higgs S (2016). Parental modelling and prompting effects on acceptance of a novel fruit in 2-4-year-old children are dependent on children’s food responsiveness. Br J Nutr.

[23] Harris HA, Fildes A, Mallan KM, et al. Maternal feeding practices and fussy eating in toddlerhood: a discordant twin analysis. Int J Behav Nutr Phys Act. 2016. 10.1186/s12966-016-0408-4. 10.1186/s12966-016-0408-4 PMC4944306 27412445

[24] Horn MG, Galloway AT, Webb RM, Gagnon SG (2011). The role of child temperament in parental child feeding practices and attitudes using a sibling design. Appetite.

[25] Larsen JK, Hermans RC, Sleddens EF, Engels RC, Fisher JO, Kremers SP (2015). How parental dietary behavior and food parenting practices affect children's dietary behavior. Interacting sources of influence?. Appetite.

[26] Corsini N, Danthiir V, Kettler L, Wilson C (2008). Factor structure and psychometric properties of the child feeding questionnaire in Australian preschool children. Appetite.

[27] Vollmer RL, Adamsons K, Foster JS, Mobley AR (2015). Association of fathers' feeding practices and feeding style on preschool age children's diet quality, eating behavior and body mass index. Appetite.

[28] Lora KR, Hubbs-Tait L, Ferris AM, Wakefield D (2016). African-American and Hispanic children's beverage intake: Differences in associations with desire to drink, fathers’ feeding practices, and weight concerns. Appetite.

[29] Hodder RK, O'Brien KM, Tzelepis F, Wyse RJ, Wolfenden L (2020). Interventions for increasing fruit and vegetable consumption in children aged five years and under. Cochrane Database Syst Rev.

[30] Adair LS, Popkin BM (2005). Are child eating patterns being transformed globally?. Obes Res.

[31] Jarman M, Ogden J, Inskip H (2015). How do mothers manage their preschool children's eating habits and does this change as children grow older? A longitudinal analysis. Appetite..

[32] McGowan L, Croker H, Wardle J, Cooke LJ (2012). Environmental and individual determinants of core and non-core food and drink intake in preschool-aged children in the United Kingdom. Eur J Clin Nutr.

[33] Early Years Analysis and Research, Department for Education Childcare and Early Years Survey of Parents in England, 2017 https://assets.publishing.service.gov.uk/government/uploads/system/uploads/attachment_data/file/669857/SFR73_2017_Text.pdf

[34] Department of Education and Training. Early childhood and child care in Summary. September quarter 2017. Canberra Australia. Available from URL: https://www.dese.gov.au/old-link-notice//system/files/doc/other/eccc_in_summary_sep_quarter_2017.pdf

[35] Statistics Canada, “Early Learning and Child Care for Children aged 0 to 5 years: A Provincial/Territorial Portrait,” https://www150.statcan.gc.ca/n1/pub/11-626-x/11-626-x2019013-eng.htm

[36] Statista. Total number of 3-and 4-year-old children enrolled in state pre-kindergarten programs in the United States in 2020, by state. https://www.statista.com/statistics/315100/total-number-of-us-children-enrolled-in-pre-k-by-state/

[37] Larson N, Ward DS, Neelon SB, Story M (2011). What role can child-care settings play in obesity prevention? A review of the evidence and call for research efforts. J Am Diet Assoc.

[38] Matwiejczyk L, Mehta K, Coveney J. Factors influencing food service provision decisions in Centre-based early childhood education and care services: Cooks’ perspective. Health Promot J Australia. 2019. 10.1002/hpja.308. 10.1002/hpja.308 31724778

[39] Adams J. Cutting children’s exposure to unhealthy food advertisements. Evidence Briefing. Available from: https://esrc.ukri.org/files/news-events-and-publications/evidence-briefings/cutting-children-s-exposure-to-unhealthy-food-advertisements/#:~:text=Ofcom%20has%20introduced%20regulations%20banning,same%20as%20before%20the%20ban. Accessed 12/08/2021.

[40] WHO (2017). Taxes on sugary drinks: Why do it?.

[41] Arksey H, O'Malley L (2005). Scoping studies: towards a methodological framework. Int J Soc Res Methodol.

[42] Levac D, Colquhoun H, O’Brien KK (2010). Scoping studies: advancing the methodology. Implementation Sci.

