# Supplementary material for: Influences on the dietary intakes of preschool children: a systematic scoping review
Source: Int J Behav Nutr Phys Act. 2022 Feb 22;19:20. doi: 10.1186/s12966-022-01254-8 (PMC8862251; doi:10.1186/s12966-022-01254-8)
Supplement: Supplementary file 2 — Additional file 2. [file 12966_2022_1254_MOESM2_ESM.docx]

**Scoping Review Protocol**

Title:

Influences on dietary intakes of preschool children

Objective:

The objective of this review is to understand the factors which are known to influence the dietary intakes of preschool children in developed countries. This will allow identification of gaps in the literature as well as factors which may be amenable to change through intervention.

Databases

Medline, Embase, Scopus, Web of Knowledge, PsychINFO, the Cochrane Library, OpenAire, EThOS, and Proquest

Search terms

Population descriptors:

(child*.OR.p$ediatric.OR.preschool*OR.age*-2-year*.OR.age*-3-year*.OR.age*-4-year*.OR.age*-5-year*)

Outcome descriptors:

(diet*.OR.diet-quality.OR.eating.OR.fruit.OR.vegetable.OR.sugar-sweetened-beverage*.OR.high-fat-salt-sugar.OR.junk-food.OR.fast-food)

| Inclusion | Exclusion |
| --- | --- |
| Population under primary-school age (<5-7 years depending on country), and over 2 years | Population attending full-time schooling |
| Outcome is solid food or drink not designed for babies/weaning | Outcome is food/drink designed for babies/weaning stage |
| Free-living population without a clinical condition | Children with a clinical condition e.g. children with ADHD. Not including obesity |
| The outcome (dependent) variable must be food/drink/energy intake. | Dietary intake is not an outcome (dependent) variable |
| Studies in developed countries (as defined by United Nations 2019) | Studies conducted developing countries (as defined by United Nations 2019) |
| Studies conducted 2000-present | Studies conducted before 2000 |
| Articles published in English | Articles written in a language other than English |

Final search strategy:

(**TS**=((child* OR preschool* OR age*-2-year* OR age*-3-year* OR age*-4-year* OR age*-5-year* OR girl OR boy)) **AND TS**=((diet* OR diet-quality OR eating OR fruit OR vegetable OR sugar-sweetened-beverage* OR high-fat-salt-sugar OR junk-food OR fast-food)) **NOT TS**=((autism OR allergy OR attention-deficit OR hyperactivity OR eating-disorder OR avoid*-restrictive-food-intake-disorder OR complimentary-feeding OR weaning OR teenage OR adolesc* OR diabetes OR anemia OR school OR kindergar*)))  *AND***LANGUAGE:** (English) *AND* **DOCUMENT TYPES:** (Article OR Book Chapter OR Data Paper OR Database Review OR Discussion OR Early Access OR Review)
