# Supplementary material for: Influences on the dietary intakes of preschool children: a systematic scoping review
Source: Int J Behav Nutr Phys Act. 2022 Feb 22;19:20. doi: 10.1186/s12966-022-01254-8 (PMC8862251; doi:10.1186/s12966-022-01254-8)
Supplement: Supplementary file 3 — Additional file 3. [file 12966_2022_1254_MOESM3_ESM.docx]

| **Reference Number** | **Title** | **First Author, Publication Year** | **Study Design** | **Study Location** | **Sample size** | **Participant** | **Child Age** | **Dietary Outcome** |
| --- | --- | --- | --- | --- | --- | --- | --- | --- |
| 1 | Specific social influences on the acceptance of novel foods in 2–5-year-old children | Addessi, 2005 | Experimental | USA | 27 | Children | 2-5 years | Novel food intake |
| 2 | The root of the problem: increasing root vegetable intake in preschool children by repeated exposure and flavour flavour learning | Ahern, 2014 | Experimental | UK | 39 | Children | 1-5 years | Vegetable puree intake |
| 3 | The effects of repeated exposure and variety on vegetable intake in pre-school children | Ahern, 2019 | Experimental | UK | 95 | Children | 2-5 years | Target vegetable intake |
| 4 | Interrelationships of More Healthful and Less Healthful Aspects of Diet Quality in a Low-Income Community Sample of Preschool Aged Children | Anderson, 2015 | Cross-sectional | USA | 298 | Caregivers | 2-5 years | Healthy food ( Fruit and vegetable , milk) and unhealthy food (sugar-sweetened beverage, fast food, sweets, salty snack) intake |
| 5 | Repeated exposure and associative conditioning promote preschool children’s liking of vegetables  Study 1 | Anzman-Frasca, 2012 | Experimental | USA | 47 | Children | 3-6 years | Vegetable intake |
| 6 | Authoritative parent feeding style is associated with better child dietary quality at dinner among low-income minority families | Arlinghaus, 2018 | Cross-sectional | USA | 275 | Caregivers | 4-5 years | Dietary quality |
| 7 | Eating in the Absence of Hunger and Weight Gain in Low-income Toddlers | Asta, 2016 | Longitudinal | USA | 209 | Mother-child pairs | 21, 27, 33 months | Palatable food intake in the absence of hunger |
| 8 | The use of inappropriate feeding practices by rural parents and their effect on preschoolers’ fruit and vegetable preferences and intake | Bante, 2008 | Cross-sectional | USA | 1555 | Parents | 2-5 years | Fruit and vegetable intake |
| 9 | The Association between Australian Childcare Centre Healthy Eating Practices and Children’s Healthy Eating Behaviours: A Cross-Sectional Study within Lunchbox Centres | Barnes, 2021 | Cross-sectional | Australia | 448 | Children | 2-5 years | Fruit and vegetable , added sugar, salt, and saturated fat intake |
| 10 | Child and family characteristics are associated with a dietary variety index in 4-year-old children from the Generation XXI cohort | Barros, 2019 | Cross-sectional | Portugal | 123 | Mothers | 4 years | Dietary variety and adequacy |
| 11 | Randomized Controlled Trial of a Clinic-Based Intervention to Promote Healthy Beverage Consumption Among Latino Children | Beck, 2017 | Intervention (RCT) | USA | 82 | Parents | 6 months – 5 years | Beverage intake |
| 12 | Short-term vegetable intake by young children classified by 6-n-propylthoiuracil bitter-taste phenotype | Bell, 2006 | Experimental | USA | 65 | Children | 3.5 – 4.5 years | Vegetable intake |
| 13 | Impact of a nutrition award scheme on the food and nutrient intakes of 2- to 4-year-olds attending long day care | Bell, 2015 | Intervention | Australia | 232 | Children | 2-4 years | Dietary intake |
| 14 | The family child-care, home environment and children's diet quality | Benjamin-Neelon, 2018 | Cross-sectional | USA | 496 | Children | 1.5 – 4 years | Dietary quality |
| 15 | Parental monitoring may protect impulsive children from overeating | Bennett, 2017 | Observational | UK | 95 | Parent-child pairs | 2-4 years | Calorie intake |
| 16 | Mothers’ child-feeding practices influence daughters’ eating and weight | Birch, 2000 | Cross-sectional | USA | 197 | Mothers | 5 years | Energy intake |
| 17 | Inducing preschool children’s emotional eating: relations with parental feeding practices | Blissett, 2010 | Experimental | UK | 25 | Mother-child pairs | 3-5 years | Calorie intake of snack foods |
| 18 | Parental modelling and prompting effects on acceptance of a novel fruit in 2–4-year-old children are dependent on children’s food responsiveness | Blissett, 2016 | Experimental | UK | 120 | Caregiver-child pairs | 2-4 years | Novel food intake |
| 19 | Home food availability and child intake among rural families identified to be at-risk for health disparities | Boles, 2019 | Cross-sectional | USA | 164 | Parents | 2-5 years | Dietary Intake |
| 20 | Managing young children’s snack food intake. The role of parenting style and feeding strategies | Boots, 2015 | Cross-sectional | Australia | 611 | Mothers | 2-7 years | Snack food intake |
| 21 | Maternal responses to difficult food request scenarios: Relationships with feeding style and child unhealthy snack intake | Boots, 2018 | Cross-sectional | Australia | 611 | Mothers | 2-7 years | Snack food intake |
| 22 | Mealtime Behaviors and Food Consumption of Perceived Picky and Non-picky Eaters through Home Use Test | Boquin, 2014 | Observational | USA | 171 | Parents | 2-4 years | Food intake at mealtime |
| 23 | Breastfeeding to 12 mo and beyond: nutrition outcomes at 3 to 5 y of age | Borkhoff, 2018 | Cross-sectional | Canada | 2987 | Mothers | 3-5 years | Dietary Intake |
| 24 | Associations between adult attachment style, emotion regulation, and preschool children’s food consumption | Bost, 2014 | Cross-sectional | USA | 497 | Parents | 2.5-3.5 years | Dietary intake |
| 25 | ‘Just a pinch of salt’. An experimental comparison of the effect of repeated exposure and flavor-flavor learning with salt or spice on vegetable acceptance in toddlers | Bouhlal, 2014 | Experimental | France | 151 | Children | 2-3 years | Vegetable intake |
| 26 | Shape of snack foods does not predict snack intake in a sample of preschoolers: a cross-over study | Boyer, 2012 | Intervention (cross-over) | USA | 28 | Children | 2-5 years | Snack food intake |
| 27 | Snack Consumption and Waste by Preschool Children Served “Cute” versus Regular Snacks | Branen, 2002 | Experimental | USA | 39 | Children | 3-5 years | Snack food intake |
| 28 | Association of Picky Eating with Weight Status and Dietary Quality Among Low-Income Preschoolers | Brown, 2018 | Cross-sectional | USA | 506 | Mothers | 3-4 years | Dietary quality and micronutrient intake |
| 29 | Parent food purchases as a measure of exposure and preschool-aged children’s willingness to identify and taste fruit and vegetables | Busick, 2008 | Cross-sectional | USA | 62 | Parents | 2-5 years | Fruit and vegetable intake |
| 30 | Preschool Children’s Acceptance of a Novel Vegetable Following Exposure to Messages in a Storybook | Byrne, 2002 | Experimental | USA | 118 | Children | 3-5 years | Novel vegetable intake |
| 31 | Beverage intake of Australian children and relationship with intake of fruit, vegetables, milk and body weight at 2, 3.7 and 5 years of age | Byrne, 2018 | Longitudinal | Australia | 2 years N= 515  3.7 years N= 426  5 years N= 405 | Mothers | 2, 3.7 and 5 years | Food & beverage intake |
| 32 | Associative Conditioning Can Increase Liking for and Consumption of Brussels Sprouts in Children Aged 3 to 5 Years | Capaldi-Philips, 2014 | Experimental | USA | 39 | Children | 3-5 years | Vegetable intake |
| 33 | Revisiting the picky eater phenomenon: Neophobic behaviors of young children | Carruth, 2000 | Longitudinal | USA | 71 | Mothers | 3.5, 5, 6 and 7 years | Dietary intake |
| 34 | Single Nucleotide Polymorphisms in Taste Receptor Genes Are Associated with Snacking Patterns of Preschool-Aged Children in the Guelph Family Health Study: A Pilot Study | Chamoun, 2018 | Cross-sectional | Canada | 47 | Children | 1.5-5 years | Snack food intake |
| 35 | Beverage Intake and Its Effect on Body Weight Status among WIC Preschool-Age Children | Charvet, 2019 | Corss-sectional | USA | 197 | Caregivers | 3-4 years | Beverage intake |
| 36 | Assessing Preschoolers’ Beverage Consumption Using the Theory of Planned Behavior | Choy, 2018 | Cross-sectional | USA | 37 | Parents | 1-5 years | Beverage intake |
| 37 | The Predictors of Diet Quality among Australian Children Aged 3.5 Years | Collins, 2016 | Longitudinal | 2016 | 248 | Mothers | 3.5 years | Diet quality |
| 38 | Relationship between parental report of food neophobia and everyday food consumption in 2 – 6-year-old children | Cooke, 2003 | Intervention (cluster-randomised trial) | UK | 564 | Mothers | 2-6 years | Dietary intake |
| 39 | Demographic, familial and trait predictors of fruit and vegetable consumption by pre-school children | Cooke, 2004 | Cross-sectional | UK | 564 | Caregivers | 2-6 years | Fruit and vegetable intake |
| 40 | Eating for Pleasure or Profit: The Effect of Incentives on Children’s Enjoyment of Vegetables | Cooke, 2011 | Experimental | UK | 422 | Children | 4-6 years | Vegetable intake |
| 41 | Pairing Vegetables with a Liked Food and Visually Appealing Presentation: Promising Strategies for Increasing Vegetable Consumption among Preschoolers | Correia, 2014 | Experimental | USA | 43 | Children | 3-5 years | Willingness to taste and total food intake |
| 42 | Parental feeding practices to manage snack food intake: Associations with energy intake regulation in young children | Corsini, 2018 | Experimental | Australia | 62 | Parents | 22-36 months | Snack intake in the absence of hunger |
| 43 | Fruit and vegetable consumption in children and their mothers. Moderating effects of child sensory sensitivity | Coulthard, 2009 | Cross-sectional | UK | 73 | Mothers | 2-5 years | Fruit and vegetable intake |
| 44 | Enjoyment of Tactile Play Is Associated with Lower Food Neophobia in Preschool Children | Coulthard, 2015 | Cross-sectional | UK | 70 | Parents | 2-5 years | Fruit and vegetable intake |
| 45 | Evaluation of a pilot sensory play intervention to increase fruit acceptance in preschool children | Coulthard, 2018 | Pilot intervention | UK | 83 | Children | 1-4 years | Fruit and vegetable intake and enjoyment |
| 46 | Feeding Strategies Derived from Behavioral Economics and Psychology Can Increase Vegetable Intake in Children as Part of a Home-Based Intervention: Results of a Pilot Study | Cravener, 2015 | Intervention (RCT) | USA | 24 | Children | 3-5 years | Vegetable and granola bar intake |
| 47 | What maternal factors influence the diet of 2-year-old children living in deprived areas? A cross-sectional survey | Crombie, 2009 | Cross-sectional | UK | 300 | Mothers | 2 years | Dietary quality |
| 48 | Child-targeted fast-food television advertising exposure is linked with fast-food intake among pre-school children | Dalton, 2017 | Cross-sectional | USA | 548 | Parents | 3-5 years | Fast-food intake |
| 49 | Positive impact of a pre-school-based nutritional intervention on children’s fruit and vegetable intake: results of a cluster-randomized trial | de Bock, 2012 | Intervention (cluster-randomised) | Germany | 348 | Children | 3-6 years | Fruit and vegetable , water & sugar-sweetened beverage intake |
| 50 | The influence of early feeding practices on fruit and vegetable intake among preschool children in 4 European birth cohorts | de Lauzon-Guillain, 2013 | Longitudinal | Europe | 9927 | Mothers | 2-4 years | Fruit and vegetable intake |
| 51 | Effectiveness of flavour nutrient learning and mere exposure as mechanisms to increase toddler’s intake and preference for green vegetable | de Wild, 2013 | Intervention (randomised) | Netherlands | 28 | Children | 21-46 months | Soup intake |
| 52 | Efficacy of repeated exposure and flavour–flavour learning as mechanisms to increase preschooler’s vegetable intake and acceptance | de Wild, 2015 | Experimental | Netherlands | 39 | Children | 1.5-4 years | Food intake |
| 53 | Use of Different Vegetable Products to Increase Preschool-Aged Children’s Preference for and Intake of a Target Vegetable: A Randomized Controlled Trial | de Wild, 2017 | Intervention (RCT) | Netherlands | 104 | Children | 2-4 years | Food intake |
| 54 | The Pattern of Complementary Foods in American Infants and Children Aged 0–5 Years Old—A Cross-Sectional Analysis of Data from the NHANES 2011–2014 | Demmer, 2018 | Cross-sectional | USA | 2431 | Caregivers | 0-5 years | Dietary intake |
| 55 | Parental reward-based eating drive predicts parents’ feeding behaviors and Children’s ultra-processed food intake | Dolwick, 2021 | Cross-sectional | USA | 190 | Parents | 3-7 years | Ultra-processed food intake |
| 56 | Preschool children’s eating behaviours are related to dietary adequacy and body weight | Dubois, 2007 | Longitudinal | Canada | 2103 | Mothers | 2.5-4.5 years | Intakes of food, energy and macronutrients |
| 57 | Effect of a Low-Intensity Parent-Focused Nutrition Intervention on Dietary Intake of 2- to 5-Year old’s | Duncanson, 2013 | Intervention (RCT) | Australia | 146 | Parents | 2-5 years | Energy intake |
| 58 | Maternal child-feeding practices and dietary inadequacy of 4-year-old children | Durao, 2015 | Cross-sectional | Portugal | 4122 | Mothers | 4 years | Dietary adequacy |
| 59 | Prompts to eat novel and familiar fruits and vegetables in families with 1e3 year-old children: Relationships with food acceptance and intake | Edelson, 2016 | Cross-sectional | USA | 60 | Parents | 1-3 years | Fruit and vegetable intake |
| 60 | Mindful feeding and child dietary health | Emley, 2017 | Cross-sectional | USA | 497 | Parents | 3-7 years | Dietary intake |
| 61 | Randomized Exposure to Food Advertisements and Eating in the Absence of Hunger Among Preschoolers | Emond, 2016 | Experimental | USA | 60 | Children | 2-5 years | Snack intake |
| 62 | Influence of child-targeted fast food TV advertising exposure on fast food intake: A longitudinal study of preschool-age children | Emond, 2019 | Longitudinal | USA | 624 | Parents | 3-5 years | Fast food intake |
| 63 | Exposure to Child-Directed TV Advertising and Preschoolers’ Intake of Advertised Cereals | Emond, 2019 | Longitudinal | USA | 624 | Parents | 3-5 years | High-sugar breakfast cereal intake |
| 64 | Ultra-Processed Food Intake and Associations with Demographic Factors in Young New Zealand Children | Fangupo, 2021 | Longitudinal | New Zealand | 669 | Mothers | 1-5 years | Ultra-processed food intake |
| 65 | Using repeated visual exposure, rewards and modelling in a mobile application to increase vegetable acceptance in children | Farrow, 2019 | Experimental | UK | 74 | Children | 3-6 years | Vegetable intake |
| 66 | Predictors of Dietary Energy Density among Preschool Aged Children | Fernando, 2018 | Cross-sectional | Australia | 209 | Mothers | 3.5 years | Dietary energy density |
| 67 | Parent-Administered Exposure to Increase Children’s Vegetable Acceptance: A Randomized Controlled Trial | Fildes, 2014 | Intervention (RCT) | UK | 442 | Children (twins) | 3-4 years | Target vegetable intake |
| 68 | Restricting Access to Foods and Children’s Eating | Fisher, 2000 | Observational | USA | 71 | Parent-child pairs | 3-5 years | Snack food intake |
| 69 | Parents' restrictive feeding practices are associated with young girls' negative self-evaluation of eating | Fisher, 2000 | Cross-sectional | USA | 197 | Parents and daughters | 4-6 years | Snack food intake |
| 70 | Parental influences on young girls’ fruit and vegetable, micronutrient, and fat intakes | Fisher, 2002 | Cross-sectional | USA | 191 | Parents | 5 years | Fruit and vegetable , energy & micronutrient intake |
| 71 | Offering “Dip” Promotes Intake of a Moderately Liked Raw Vegetable among Preschoolers with Genetic Sensitivity to Bitterness | Fisher, 2012 | Experimental | USA | 152 | Children | 3-5 years | Broccoli intake |
| 72 | Influences on the quality of young children's diets: the importance of maternal food choices | Fisk, 2011 | Cross-sectional | UK | 1640 | Mothers | 3 years | Diet quality |
| 73 | Playing with food: The effects of food pre-exposure on consumption in young children | Florack, 2018 | Experimental | Not specified | 81 | Children | 3-6 years | Food intake |
| 74 | A description of an ‘obesogenic’ eating style that promotes higher energy intake and is associated with greater adiposity in 4.5-year-old children: Results from the GUSTO cohort | Fogel, 2017 | Observational | Singapore | 386 | Parents and children | 4.5 years | Energy intakes |
| 75 | Oral processing behaviours that promote children's energy intake are associated with parent-reported appetitive traits: Results from the GUSTO cohort | Fogel, 2018 | Longitudinal | Singapore | 195 | Mothers | 4.5 and 6 years | Energy intake |
| 76 | Eating behaviors moderate the associations between risk factors in the first 1000 days and adiposity outcomes at 6 years of age | Fogel, 2020 | Observational | Singapore | 302 | Children | 6 years | Energy intake |
| 77 | Does Eating during Television Viewing Affect Preschool Children’s Intake? | Francis, 2006 | Experimental | USA | 24 | Children | 3-5 years | Food intake |
| 78 | Maternal feeding practices and children's food intake during an ad libitum buffet meal: Results from the GUSTO cohort | Fries, 2019 | Observational | Singapore | 201 | Mothers | 4.5 years | Energy intake |
| 79 | Predicting preschool children's eating in the absence of hunger from maternal pressure to eat: A longitudinal study of low-income, Latina mothers | Galindo, 2018 | Longitudinal | USA | 138 | Mothers | 4-5 years | Calorie intake in the absence of hunger |
| 80 | ‘Finish your soup’: Counterproductive effects of pressuring children to eat on intake and affect | Galloway, 2006 | Experimental | USA | 27 | Children | 3-5 years | Soup intake |
| 81 | Consumption of soda and other sugar-sweetened beverages by 2-year-olds: findings from a population-based survey | Garnett, 2013 | Cross-sectional | USA | 1911 | Mothers | 2 years | Sugar-sweetened beverage Intake |
| 82 | Vegetable/Fruit Intakes of Young Children at Home and in Childcare Centres | Gatien, 2020 | Cross-sectional | Canada | 79 | Children | 3-5 years | Fruit and vegetable intake |
| 83 | Delay of Gratification Predicts Eating in the Absence of Hunger in Preschool-Aged Children | Giuliani, 2021 | Longitudinal | USA | 47 | Mothers and children | 3-6 years | Snack intake in the absence of hunger |
| 84 | The role of family variables in fruit and vegetable consumption in pre-school children | Goldman, 2012 | Cross-sectional | USA | 229 | Caregivers | 2-5 years | Dietary intake |
| 85 | Parental Information, Motivation, and Behavioral Skills Correlate with Child Sweetened Beverage Consumption | Goodell, 2012 | Cross-sectional | USA | 198 | Parents | 1-5 years | Calorie intake from sugar-sweetened beverage |
| 86 | Positive- and negative peer modelling effects on young children’s consumption of novel blue foods (Study 2) | Greenhalgh, 2009 | Experimental | UK | 49 | Children | 3-4 years | Novel food intake |
| 87 | Maternal feeding practices predict fruit and vegetable consumption in young children. Results of a 12-month longitudinal study | Gregory, 2011 | Longitudinal | Australia | 60 | Mothers | 2 years | Fruit and vegetable and sweet food intake |
| 88 | Teaching Young Children a Theory of Nutrition: Conceptual Change and the Potential for Increased Vegetable Consumption (Study 1 &2) | Gripshover, 2013 | Experimental | USA | 1) 59  2) 103 | Children | 4-5 years | Number of snack foods eaten |
| 89 | Clustering of Dietary Intake and Sedentary Behavior in 2-Year-Old Children | Gubbels, 2009 | Cross-sectional | Netherlands | 2578 | Mothers | 2 years | Dietary intake |
| 90 | Diet-related restrictive parenting practices. Impact on dietary intake of 2-year-old children and interactions with child characteristic | Gubbels, 2009 | Cross-sectional | Netherlands | 2578 | Mothers | 2 years | Dietary intake |
| 91 | Child-care environment and dietary intake of 2- and 3-year-old children | Gubbels, 2010 | Cross-sectional | Netherlands | 135 | Children | 2-3 years | Dietary intake |
| 92 | Dietary Intake by Dutch 1- to 3-Year-Old Children at Childcare and at Home | Gubbels, 2014 | Cross-sectional | Netherlands | 1016 | Childcare staff and parents | 1-3 years | Dietary intake |
| 93 | High 5 for Kids: The impact of a home visiting program on fruit and vegetable intake of parents and their preschool children | Haire-Joshu, 2008 | Intervention | USA | 1658 | Parents | 2-5 years | Fruit and vegetable intake |
| 94 | Associations Between Swedish Mothers’ and 3- and 5-Year-Old Children’s Food Intake | Hansson, 2016 | Cross-sectional | Sweden | 189 | Mothers | 3 and 5 years | Fruit and vegetable intake |
| 95 | The relationship between controlling feeding practices and boys' and girls' eating in the absence of hunger | Harris, 2014 | Experimental | Australia | 37 | Mother-child pairs | 3-4 years | Eating in the absence of hunger (snack intake) |
| 96 | Mere exposure and flavour–flavour learning increase 2–3 year-old children’s acceptance of a novel vegetable | Hausner, 2012 | Experimental | Denmark | 104 | Children | 2-3 years | Vegetable puree intake |
| 97 | Parenting Self-Efficacy, Parent Depression, and Healthy Childhood Behaviors in a Low-Income Minority Population: A Cross-Sectional Analysis | Heerman, 2017 | Cross-sectional | USA | 601 | Caregivers | 3-5 years | Dietary intake |
| 98 | Effectiveness of trained peer models to encourage food acceptance in preschool children | Hendy, 2002 | Experimental | USA | 38 | Children | 3-6 years | Novel food intake |
| 99 | Associations among parental feeding styles and children's food intake in families with limited incomes | Hoerr, 2009 | Cross-sectional | USA | 715 | Caregivers | Child age NR | Dietary intake |
| 100 | ‘Why don’t you try it again?’ A comparison of parent led, home based interventions aimed at increasing children’s consumption of a disliked vegetable | Holley, 2015 | Experimental | UK | 115 | Parent-child pairs | 2-4 years | Target vegetable intake |
| 101 | Investigating the role of parent and child characteristics in healthy eating intervention outcomes | Holley, 2016 | Experimental | UK | 90 | Parent-child pairs | 2-4 years | Target vegetable intake |
| 102 | Predicting children’s fussiness with vegetables: The role of feeding practices | Holley, 2018 | Cross-sectional | UK | 297 | Caregivers | 19-62 months | Vegetable intake |
| 103 | The hTAS2R38 genotype is associated with sugar and candy consumption in preschool boys | Hoppu, 2015 | Cross-sectional | Finland | 400 | Children | 2-6 years | Dietary intake |
| 104 | Increasing pre-school children’s consumption of fruit and vegetables. A modelling and rewards intervention | Horne, 2011 | Intervention (baseline to outcome changes) | UK | 20 | Children | 2-4 years | Fruit and vegetable intake |
| 105 | Low-Income African American and Non-Hispanic White Mothers’ Self-Efficacy, ‘‘Picky Eater’’ Perception, and Toddler Fruit and Vegetable Consumption | Horodynski, 2010 | Cross-sectional | USA | 399 | Mothers | 1-3 years | Fruit and vegetable intake |
| 106 | Adversity exposure and obesogenic food consumption in young children: The transgenerational role of emotion dysregulation | Huffhines, 2020 | Longitudinal | USA | 190 | Caregivers | 3-5 years | Obesogenic food intake |
| 107 | The Impact of Child Care Providers’ Feeding on Children’s Food Consumption | Hughes, 2007 | Observational | USA | 549 | Children | 3-5 years | Dietary intake |
| 108 | Parental feeding styles, young children’s fruit, vegetable, water and sugar-sweetened beverage consumption, and the moderating role of maternal education and ethnic background | Inhulsen, 2017 | Cross-sectional | Netherlands | 5926 | Parents | 3-7 years | Dietary intake |
| 109 | Obesogenic food consumption among young children: the role of maltreatment | Jackson, 2019 | Longitudinal | USA | 4800 | Mothers | 3-5 years | Obesogenic food intake |
| 110 | Location influences snacking behavior of US infants, toddlers and preschool children | Jacquier, 2018 | Cross-sectional | USA | 1461 | Parents | 2-4 years | Dietary intake |
| 111 | Relationships Among Parental Psychological Distress, Parental Feeding Practices, Child Diet, and Child Body Mass Index | Jang, 2019 | Cross-sectional | USA | 256 | Caregivers | 2-5 years | Dietary intake |
| 112 | Association between Australian-Indian mothers’ controlling feeding practices and children’s appetite traits | Jani, 2015 | Cross-sectional | Australia | 203 | Mothers | 1-5 years | Diet quality |
| 113 | Associations between Sleep and Dietary Patterns among Low-Income Children Attending Preschool | Jansen, 2019 | Cross-sectional | USA | 350 | Parents | 3-5 years | Dietary patterns |
| 114 | Influences on the diet quality of pre-school children: importance of maternal psychological characteristics | Jarman, 2014 | Cross-sectional | UK | 324 | Mothers | 2-4 years | Diet quality |
| 115 | How do mothers manage their preschool children's eating habits and does this change as children grow older? A longitudinal analysis | Jarman, 2015 | Longitudinal | UK | 228 | Mothers | 2-5 years | Diet quality |
| 116 | Maternal prompting types and child vegetable intake: Exploring the moderating role of picky eating | Jordan, 2020 | Observational | USA | 199 | Caregivers | *M* = 6 years | Vegetable intake |
| 117 | Sensory-based food education in early childhood education and care, willingness to choose and eat fruit and vegetables, and the moderating role of maternal education and food neophobia | Kahkonen, 2018 | Cross-sectional | Finland | 130 | Parents and children | 3-5 years | Fruit and vegetable intake |
| 118 | A cross-sectional study of children's temperament, food consumption and the role of food-related parenting practices | Kaukonen, 2019 | Cross-sectional | Finland | 864 | Parents | 3-6 years | Food intake |
| 119 | Inherited Taste Sensitivity to 6-n-Propylthiouracilin Diet and Body Weight in Children | Keller, 2004 | Cross-sectional | USA | 53 | Mothers and children | 4-5 years | Dietary intake |
| 120 | Increased Sweetened Beverage Intake Is Associated with Reduced Milk and Calcium Intake in 3- to 7-Year-Old Children at Multi-Item Laboratory Lunches | Keller, 2009 | Cross-sectional | USA | 126 | Children (twins) | 3-7 years | Beverage and calcium intake |
| 121 | The impact of food branding on children's eating behavior and obesity  Study 3 | Keller, 2012 | Pilot intervention | USA | 16 | Children | 4-5 years | Fruit and vegetable intake |
| 122 | Food consumption by young children: A function of parental feeding goals and practices | Kiefner-Burmeister, 2014 | Cross-sectional | USA | 171 | Mothers | 3-6 years | Dietary intake |
| 123 | Do drinking buddies matter for young children?: Preschoolers’ conformity to remote peers’ beverage choices | Kim, 2020 | Experimental | Singapore | 63 | Children | 3-6 years | Beverage intake |
| 124 | Double trouble: Portion size and energy density combine to increase preschool children's lunch intake | Kling, 2016 | Experimental | USA | 120 | Children | 3-5 years | Food and energy intake |
| 125 | Two-Year Outcomes of the Enabling Mothers to Prevent Pediatric Obesity Through Web-Based Education and Reciprocal Determinism (EMPOWER) Randomized Control Trial | Knowlden, 2018 | Intervention (RCT) | USA | 57 | Mothers | 4-6 years | Fruit and vegetable and sugar-free beverage intake |
| 126 | Associations of mothers' source of feeding information with longitudinal trajectories of sugar-sweetened beverage intake, 100% juice intake and adiposity in early childhood | Korn, 2021 | Longitudinal | USA | 371 | Mothers | 3-7 years | Beverage intake |
| 127 | The Influence of Media Characters on Children’s Food Choices | Kotler, 2012 | Experimental | USA | 207 | Children | 3-6 years | Food intake |
| 128 | Dietary patterns among Norwegian 2-year-olds in 1999 and in 2007 and associations with child and parent characteristics | Kristiansen, 2013 | Cross-sectional | Norway | 1472 | Mothers | 2 years | Dietary patterns |
| 129 | Maternal and child dietary intake: The role of maternal healthy-eater self-schema | Kueppers, 2018 | Cross-sectional | USA | 124 | Mothers | 2-5 years | Fruit and vegetable, saturated fat and added sugar intake |
| 130 | Mother–child dietary behaviours and their observed associations with socio-demographic factors: ﬁndings from the Healthy Beginnings Trial | Kunaratnam, 2018 | Cross-sectional | Australia | 243 | Mothers | 2 years | Dietary pattern |
| 131 | Association between hair cortisol concentration and dietary intake among normal weight preschool children predisposed to overweight and obesity | Larsen, 2019 | Cross-sectional | Denmark | 296 | Parents and children | 2-6 years | Dietary intake and quality |
| 132 | Reducing the energy density of multiple meals decreases the energy intake of preschool-age children | Leahy, 2008 | Experimental | USA | 36 | Children | 3-5 years | Food and beverage intake |
| 133 | Reductions in Entrée Energy Density Increase Children’s Vegetable Intake and Reduce Energy Intake | Leahy, 2008 | Experimental | USA | 61 | Children | 3-5 years | Vegetable and energy intake |
| 134 | Obesogenic home food availability, diet, and BMI in Pakistani and White toddlers | LeCroy, 2020 | Longitudinal | UK | 882 | Mothers | 18 months and 36 months | Snack food and SSB intake at 36 months |
| 135 | Early life determinants of dietary patterns in preschool children: Rhea mother–child cohort, Crete, Greece | Leventakou, 2016 | Longitudinal | Greece | 1601 | Mothers | 4 years | Dietary patterns |
| 136 | Dietary Intake Among Head Start Preschooler-caregiver Dyads | Ling, 2018 | Cross-sectional | USA | 70 | Parents | 3-5 years | Dietary intake |
| 137 | Dietary Patterns Track from Infancy to Preschool Age: Cross-Sectional and Longitudinal Perspectives | Lioret, 2015 | Longitudinal | France | 1899 | Mothers | 2, 3 and 5 years | Dietary patterns |
| 138 | Impact of Portion Size and Energy Density on Snack Intake in Preschool-Aged Children | Looney, 2011 | Experimental | USA | 17 | Children | 2-5 years | Food intake |
| 139 | African American and Hispanic children's beverage intake: Differences in associations with desire to drink, fathers' feeding practices, and weight concerns | Lora, 2016 | Cross-sectional | USA | 110 | Fathers | 2-5 years | Beverage intake |
| 140 | Preschoolers' influence on and help with beverage selection at the grocery store is linked to maternal responsiveness and child beverage intake: An exploratory study | Lora, 2016 | Cross-sectional | 2016 | 185 | Mothers | 3-5 years | Beverage intake |
| 141 | Home food environment factors associated with Hispanic preschoolers’ intake of fruits and vegetables | Lora, 2019 | Cross-sectional | USA | 238 | Mother-child pairs | *M* = 3.8 years | Fruit and vegetable intake |
| 142 | Contributions of Early Care and Education Programs to Diet Quality in Children Aged 3 to 4 Years in Central North Carolina | Luecking, 2019 | Cross-sectional | USA | 840 | Children | 3-4 years | Dietary quality in early care and education |
| 143 | Maternal prompts to eat, child compliance, and mother and child weight status | Lumeng, 2006 | Observational | USA | 71 | Mother-child pairs | 3-6 years | Percentage of compliance to eat prompted food |
| 144 | Unhealthy Dietary Patterns Established in Infancy Track to Mid-Childhood: The EU Childhood Obesity Project | Luque, 2018 | Longitudinal | Europe | 633 | Caregivers | 1, 2, 3, 4, 5, 6 and 8 years | Dietary pattern |
| 145 | Child Dietary and Eating Behavior Outcomes up to 3.5 Years After an Early Feeding Intervention: The NOURISH RCT | Magarey, 2016 | Intervention (RCT) | Australia | 698 | Mothers | 2, 3.7 and 5 years | Dietary intake |
| 146 | The Relationship between Number of Fruits, Vegetables, and Noncore Foods Tried at Age 14 Months and Food Preferences, Dietary Intake Patterns, Fussy Eating Behavior, and Weight Status at Age 3.7 Years | Mallan, 2015 | Longitudinal | Australia | 340 | Mothers | 3.7 years | Fruit and vegetable, and non-core food intake |
| 147 | Television viewing and food habits in toddlers and preschoolers in Greece: the GENESIS study | Manios, 2009 | Cross-sectional | Greece | 2374 | Parents | 1-5 years | Daily energy intake |
| 148 | Relationships of family conflict, cohesion, and chaos in the home environment on maternal and child food‐related behaviours | Martin-Biggers, 2018 | Cross-sectional | USA | 550 | Mothers | 2-5 years | Dietary Intake |
| 149 | Environmental and individual determinants of core and non-core food and drink intake in preschool-aged children in the United Kingdom | McGowan, 2012 | Cross-sectional | UK | 434 | Caregivers | 2-5 years | Core & non-core food intake |
| 150 | Predicting what mothers feed their preschoolers: Guided by an extended theory of planned behaviour | McKee, 2019 | Longitudinal | Australia | 235 | Mothers | 2-4 years | Fruit and vegetable, snack and drink intake |
| 151 | Maternal predictors of preschool child-eating behaviours, food intake and body mass index: a prospective study | McPhie, 2012 | Longitudinal | Australia | 117 | Mothers | 2-4 years | Fruit and vegetable, and unhealthy food intake |
| 152 | Family food involvement is related to healthier dietary intake in preschool-aged children | Metcalfe, 2018 | Cross-sectional | USA | 497 | Parents | 3 and 4 years | Dietary Intake |
| 153 | Associations between food and beverage consumption and different types of sedentary behaviours in European preschoolers: the ToyBox‑study | Miguel-Berges, 2017 | Cross-sectional | Europe | 6431 | Caregivers | 3.5-5.5 years | Food and beverage intake |
| 154 | Clustering of energy balance-related behaviours and parental education in European preschool children: the ToyBox study | Miguel-Berges, 2017 | Cross-sectional | Europe | 5387 | Parents | 3.5-5.5 years | Food and beverage intake |
| 155 | Sleep duration and quality are associated with eating behavior in low-income toddlers | Miller, 2019 | Cross-sectional | USA | 134 | Mother-child pairs | 33 months | Calorie intake in the absence of hunger |
| 156 | Guelph Family Health Study’s Home-Based Obesity Prevention Intervention Increases Fibre and Fruit Intake in Preschool-Aged Children | Mirotta, 2018 | Intervention (RCT) | Canada | 45 | Families | 1.5-5 years | Energy Intake |
| 157 | Stability of food neophobia from infancy through early childhood | Moding, 2016 | Longitudinal | USA | 115 | Mothers | 4.5 years | Novel food intake |
| 158 | Infant nutrition in relation to eating behaviour and fruit and vegetable intake at age 5 years | Moller, 2013 | Longitudinal | Netherlands | 3624 | Mothers | 5 years | Fruit and vegetable Intake |
| 159 | Eating out of home and dietary adequacy in preschool children | Moreira, 2015 | Cross-sectional | Portugal | 2414 | Mothers | Median = 51 months | Dietary intake |
| 160 | Feeding behaviors of low-income mothers: directive control relates to a lower BMI in children, and a nondirective control relates to a healthier diet in preschoolers | Murashima, 2012 | Cross-sectional | USA | 330 | Mothers | 3-5 years | Dietary intake and adequacy |
| 161 | Parental perception of child weight in the first two years-of-life: a potential link between infant feeding and preschoolers’ diet | Musaad, 2015 | Cross-sectional | USA | 497 | Parents | 2-5 years | Dietary intake |
| 162 | Emerald dragon bites vs veggie beans: Fun food names increase children's consumption of novel healthy foods | Musher-Eizenman, 2011 | Experimental | USA | 36 | Children | 1-7 years | Novel food intake |
| 163 | Watch Me Grow: A garden-based pilot intervention to increase vegetable and fruit intake in preschoolers | Namenek Brouwer, 2013 | Intervention (RCT) | USA | 4 childcare centres  number of children not reported | Children | 3-5 years | Fruit and vegetable intake |
| 164 | Mother’s perception of general family functioning and sugar consumption of 3 and 4-year-old children: the ELF study | Nanjappa, 2015 | Cross-sectional | UK | 698 | Mothers | 3 & 4 years | Sugar food and beverage intake |
| 165 | Taste Exposure Increases Intake and Nutrition Education Increases Willingness to Try an Unfamiliar Vegetable in Preschool Children: A Cluster Randomized Trial | Nekitsing, 2019 | Intervention (cluster RCT) | UK | 219 | Children | 2-5 years | Mooli intake |
| 166 | A mHealth randomized controlled trial to reduce sugar sweetened beverage intake in preschool-aged children | Nezami, 2018 | Intervention (RCT) | USA | 51 | Mothers | 3-5 years | Beverage intake |
| 167 | Motivational theater to increase consumption of vegetable dishes by preschool children | Nicklas, 2017 | Intervention (randomised feasibility) | USA | 253 | Children | *M* = 4.4 years | Vegetable intake |
| 168 | The effects of taste sensitivity and repeated taste exposure on children’s intake and liking of turnip Brassica rapa subsp. rapa); a bitter Brassica vegetable | Nor, 2021 | Experimental | UK | 172 | Children | 3-5 years | Turnip intake |
| 169 | Multivariate analysis of diet among three-year-old children and associations with socio-demographic characteristics | North, 2000 | Cross-sectional | UK | 7814 | Mothers | 3 years | Dietary patterns |
| 170 | Multivariate analysis of diet in children at four and seven years of age and associations with socio-demographic characteristics | Northstone, 2005 | Cross-sectional | UK | 9550 | Mothers | 4 years | Dietary patterns |
| 171 | The associations between feeding difficulties and behaviours and dietary patterns at 2 years of age: the ALSPAC cohort | Northstone, 2013 | Cross-sectional | UK | 9599 | Mothers | 2 years | Dietary patterns |
| 172 | Mobile-based intervention intended to stop obesity in preschool-aged children: the MINISTOP randomized controlled trial | Nystrom, 2017 | Intervention (RCT) | Sweden | 315 | Parents | 4.5 years | Fruit and vegetable, candy and sugar=sweetens beverage intake |
| 173 | Repeated Exposure in a Natural Setting: A Preschool Intervention to Increase Vegetable Consumption | O’Connell, 2012 | Intervention (RCT) | USA | 96 | Children | 3-6 years | Vegetable intake |
| 174 | Parenting practices are associated with fruit and vegetable consumption in pre-school children | O’Connor, 2010 | Cross-sectional | USA | 755 | Caregivers | 3-5 years | Dietary intake |
| 175 | The association of TV viewing during dinner meals with quality of dietary intake and BMI z-scores among low income, ethnic minority preschool children | O’Connor, 2019 | Observational | USA | 144 | Children | 3-5 years | Diet quality |
| 176 | Parental restriction and children’s diets. The chocolate coin and Easter egg experiments  Study 1 | Ogden, 2013 | Experimental | UK | 53 | Parents and children | 1-7 years | Chocolate intake |
| 177 | Parental food involvement predicts parent and child intakes of fruits and vegetables | Ohly, 2013 | Cross-sectional | UK | 394 | Parents | 1.5-5 years | Diet quality |
| 178 | Children’s bite size and intake of an entrée are greater with large portions than with age-appropriate or self-selected portions | Orlet Fisher, 2003 | Experimental | USA | 30 | Children | 4 years | Entrée intake |
| 179 | The effect of the home environment on physical activity and dietary intake in preschool children | Østbye, 2013 | Cross-sectional | USA | 208 | Mothers | 2-5 years | Healthy and junk food intake |
| 180 | Peas, please! Food familiarization through picture books helps parents introduce vegetables into preschoolers’ diets | Owen, 2018 | Intervention | UK | 127 | Children | 21-24 months | Novel fruit or vegetable intake |
| 181 | Feeding Style Differences in Food Parenting Practices Associated with Fruit and Vegetable Intake in Children from Low-income Families | Papaiannou, 2013 | Cross-sectional | USA | 667 | Parents | 4.4 years | Fruit and vegetable Intake |
| 182 | The benefits of authoritative feeding style: caregiver feeding styles and children’s food consumption patterns | Patrick, 2005 | Cross-sectional | USA | 231 | Caregivers | 3-5 years | Fruit and vegetable, and dairy intake |
| 183 | Association of TAS2R38 variants with sweet food intake in children aged 1-6 years | Pawellek, 2016 | Longitudinal | Europe | 684 | Children | 1-6 years | Energy intake from sweet foods |
| 184 | Healthy eating behaviors and the cognitive environment are positively associated in low-income households with young children | Pieper, 2011 | Cross-sectional | USA | 3645 | Parents | 1-5 years | Dietary intake |
| 185 | Dietary patterns of Australian children at three and five years of age and their changes over time: A latent class and latent transition analysis | Pitt, 2018 | Longitudinal | Australia | 1565 | Mothers | 3 and 5 years | Dietary patterns |
| 186 | Character Apps for Children’s Snacks: Effects of Character Awareness on Snack Selection and Consumption Patterns | Putnam, 2018 | Experimental | USA | 132 | Children | 4-5 years | Snack food intake |
| 187 | Maternal feeding practices in relation to dietary intakes and BMI in 5-year-olds in a multi-ethnic Asian population | Quah, 2018 | Cross-sectional | Singapore | 511 | Mothers | 5 years | Dietary intake |
| 188 | Socioeconomic characteristics of mothers and their relationship with dietary diversity and food group consumption of their children | Quezada-Sanchez, 2020 | Cross-sectional | Mexico | 1041 | Mothers | 1-4 years | Dietary diversity and intake |
| 189 | Influencing factors of children's fruit, vegetable and sugar-enriched food intake in a Finnish preschool setting: Preschool personnel's perceptions | Ray, 2016 | Cross-sectional | Finland | 14 | Preschool personnel (pre-school teachers, day care nurses) | NR | Fruit and vegetable, and sugary food intake |
| 190 | Increasing food acceptance in the home setting: a randomized controlled trial of parent-administered taste exposure with incentives | Remington, 2012 | Intervention (RCT) | UK | 149 | Children | 3-4 years | Intake of target vegetable |
| 191 | Cluster randomized control trial promoting child self-regulation around energy-dense food | Rhee, 2019 | Intervention (cluster RCT) | USA | 92 | Children | 4-6 years | Calorie intake in the absence of hunger |
| 192 | Visual exposure and categorization performance positively influence 3- to 6-year-old children's willingness to taste unfamiliar vegetables | Rioux, 2018 | Intervention | France | 70 | Children | 3-6 years | Vegetable intake |
| 193 | Influence of Licensed Characters on Children’s Taste and Snack Preferences | Roberto, 2010 | Experimental | USA | 40 | Children | 4-6 years | Food preference |
| 194 | Parent packs, child eats: Surprising results of Lunch is in the Bags efficacy trial | Roberts-Grey, 2018 | Intervention | USA | 633 | Parent-child pairs | 3-5 years | Lunch food intake |
| 195 | Serving a variety of vegetables and fruit as a snack increased intake in preschool children | Roe, 2013 | Experimental | USA | 61 | Children | 3-5 years | Fruit and vegetable intake |
| 196 | Relationship between pickiness and subsequent development in body mass index and diet intake in obesity prone normal weight preschool children | Rohde, 2017 | Longitudinal | Denmark | 271 | Parents | 2-6 years | Dietary intake |
| 197 | Cross-sectional associations between maternal self-efficacy and dietary intake and physical activity in four-year-old children of first-time Swedish mothers | Rohde, 2018 | Cross-sectional | Sweden | 249 | Mothers | 4 years | Dietary intake |
| 198 | Preschoolers will drink their GREENS! Children accept, like, and drink novel smoothies containing dark green vegetables (DGVs) | Rollins, 2021 | Experimental | USA | 68 | Children | 3-5 years | Smoothie intake |
| 199 | Predictors and Outcomes of Mealtime Emotional Climate in Families with Preschoolers | Saltzman, 2018 | Longitudinal | USA | 74 | Parents | 3 and 4 years | Dietary intake |
| 200 | Impact of parental education and income inequality on children’s food intake | Sausenthaler, 2007 | Cross-sectional | Germany | 2637 | Parents | 2 years | Dietary intake |
| 201 | Serving smaller age-appropriate entrée portions to children aged 3–5 y increases fruit and vegetable intake and reduces energy density and energy intake at lunch | Savage, 2012 | Experimental | USA | 17 | Children | 3-5 years | Energy intake |
| 202 | The Addition of a Plain or Herb-Flavored Reduced-Fat Dip Is Associated with Improved Preschoolers’ Intake of Vegetables | Savage, 2013 | Quasi-experimental | USA | 27 | Children | 3-5 years | Vegetable intake |
| 203 | Do children eat less at meals when allowed to serve themselves? | Savage, 2016 | Experimental | USA | 63 | Children | 3-5 years | Energy intake |
| 204 | The Obesogenic Quality of the Home Environment: Associations with Diet, Physical Activity, TV Viewing, and BMI in Preschool Children | Schrempft, 2015 | Cross-sectional | UK | 1096 | Caregivers | 4 years | Dietary intake |
| 205 | Fruit and Vegetable Intakes of Preschool Children Are Associated with Feeding Practices Facilitating Internalization of Extrinsic Motivation | Shim, 2016 | Cross-sectional | USA | 316 | Mothers | 2-5 years | Fruit and vegetable intake |
| 206 | Fetal growth interacts with multilocus genetic score reflecting dopamine signaling capacity to predict spontaneous sugar intake in children | Silveria, 2017 | Longitudinal | Canada | 192 | Children | 4 years | Macronutrient intake of snacks |
| 207 | Do changes in objective and subjective family income predict change in children's diets over time? Unique insights using a longitudinal cohort study and fixed effects analysis? | Skafina, 2014 | Longitudinal | UK | 3279 | Mothers | 2-5 years | Dietary intake |
| 208 | Portion size has sustained effects over 5 days in preschool children: a randomized trial | Smethers, 2019 | Experimental | USA | 46 | Children | 3-5 years | Energy intake |
| 209 | Eating vegetables first: the use of portion size to increase vegetable intake in preschool children | Spill, 2010 | Experimental | USA | 51 | Children | 3-5 years | Vegetable and energy intake |
| 210 | Hiding vegetables to reduce energy density: an effective strategy to increase children’s vegetable intake and reduce energy intake | Spill, 2011 | Experimental | USA | 40 | Children | 3-5 years | Vegetable intake |
| 211 | Serving large portions of vegetable soup at the start of a meal affected children’s energy and vegetable intake | Spill, 2011 | Experimental | USA | 72 | Children | 3-5 years | Energy, vegetable and soup intake |
| 212 | Relationships between the home environment and physical activity and dietary patterns of preschool children: a cross-sectional study | Spurrier, 2008 | Cross-sectional | Australia | 280 | Parents | 4-5 years | Dietary intake |
| 213 | Influence of Screen-Based Peer Modeling on Preschool Children’s Vegetable Consumption and Preferences | Staiano, 2016 | Intervention (RCT) | USA | 42 | Children | 3-5 years | Vegetable intake |
| 214 | Pediatric Nutrition: Parenting Impacts Beyond Financial Resources | Swindle, 2014 | Cross-sectional | USA | 446 | Parents | 2-5 years | Fruit and vegteable intake |
| 215 | Patterns of Complementary Feeding Behaviors Predict Diet Quality in Early Childhood | Switkowski, 2020 | Longitudinal | USA | 1162 | Mother-child pairs | 3 years | Dietary quality |
| 216 | Family Ties to Health Program: A Randomized Intervention to Improve Vegetable Intake in Children | Tabak, 2012 | Intervention (RCT) | USA | 43 | Families | 2-5 years | Vegetable intake |
| 217 | Changes in Parent Motivation Predicts Changes in Body Mass Index z-Score (zBMI) and Dietary Intake Among Preschoolers Enrolled in a Family-Based Obesity Intervention | Van-Allen, 2014 | Intervention (RCT) | USA | 42 | Families | 2-5 years | Dietary intake |
| 218 | Picky eating: Associations with child eating characteristics and food intake | Van der Horst, 2016 | Cross-sectional | USA | 2371 | Caregiver | 1-4 years | Dietary intake |
| 219 | What matters most - what parents model or what parents eat? | Vaughn, 2018 | Cross-sectional | USA | 266 | Parents | *M* = 3.4 years | Diet quality |
| 220 | Do stressed children have a lot on their plates? A cross-sectional study of long-term stress and diet among Finnish preschoolers | Vepsäläinen, 2021 | Cross-sectional | Finland | 597 | Parents and children | 3-6 years | Food intake frequencies and dietary patterns |
| 221 | Influence of mother’s educational level on food parenting practices and food habits of young children | Vereecken, 2004 | Cross-sectional | Belgium | 316 | Mothers | 2.5-7 years | Fruit and vegetable, sugar-sweetened beverage and sweet intake |
| 222 | Associations of parenting styles, parental feeding practices and child characteristics with young children’s fruit and vegetable consumption | Vereecken, 2010 | Cross-sectional | Belgium | 755 | Parents | *M* = 3.5 years | Fruit and vegetable intake |
| 223 | Association of fathers’ feeding practices and feeding style on preschool age children’s diet quality, eating behavior and body mass index | Vollmer, 2015 | Cross-sectional | USA | 150 | Fathers | 3-5 years | Diet quality |
| 224 | Investigating the Relationship of Body Mass Index, Diet Quality, and Physical Activity Level between Fathers and Their Preschool-Aged Children | Vollmer, 2015 | Cross-sectional | USA | 150 | Fathers | 3-5 years | Diet quality |
| 225 | Associations Between Temperament at Age 1.5 Years and Obesogenic Diet at Ages 3 and 7 Years | Vollrath, 2012 | Longitudinal | Norway | 6697 | Mothers | 3 and 7 years | Fruit and vegteable, sweet food and sweet drink intake |
| 226 | Dietary patterns of children at 3.5 and 7 years of age: a New Zealand birth cohort study | Wall, 2012 | Cross-sectional | New Zealand | 550 | Mothers | 3.5 years | Dietary patterns |
| 227 | Parents and Tots Together: Pilot randomized controlled trial of a family-based obesity prevention intervention in Canada | Walton, 2015 | Pilot Intervention (RCT) | Canada | 48 | Parents | 2-5 years | Sugar-sweetened beverage intake |
| 228 | Association between childcare educators’ practices and preschoolers’ physical activity and dietary intake: a cross-sectional analysis | Ward, 2017 | Observational | Canada | 732 | Children | *M* = 4 years | Dietary intake |
| 229 | “Monkey see, monkey do”: Peers' behaviors predict preschoolers' physical activity and dietary intake in childcare centers | Ward, 2017 | Longitudinal | Canada | 238 | Children | 3-5 years | Dietary intake |
| 230 | Maternal Depression: Relationship to Food Insecurity and Preschooler Fruit/Vegetable Consumption | Ward, 2020 | Cross-sectional | USA | 693 | Caregivers | 3-5 years | Fruit and vegetable intake |
| 231 | Increasing children’s acceptance of vegetables; a randomized `trial of parent-led exposure | Wardle, 2003 | Intervention (RCT) | UK | 156 | Children | 2-6 years | Target vegetable intake |
| 232 | Parental control over feeding and children’s fruit and vegetable intake: How are they related? | Wardle, 2005 | Cross-sectional | UK | 564 | Parents | 2-6 years | Fruit and vegetable intake |
| 233 | The FTO gene and measured food intake in children | Wardle, 2009 | Experimental | UK | 131 | Children | 4-5 years | Biscuit intake |
| 234 | Family stress predicts poorer dietary quality in children: Examining the role of the parent–child relationship | Webb, 2018 | Longitudinal | Australia | 579 | Mothers | 3 and 5 years | Dietary intake |
| 235 | Associations between Family Meal Context and Diet Quality among Preschool-Aged Children in the Guelph Family Health Study | Wedde, 2020 | Cross-sectional | Canada | 95 | Children | 2-5 years | Dietary quality |
| 236 | Room for Improvement Remains in Food Consumption Patterns of Young Children Aged 2–4 Years | Welker, 2018 | Cross-sectional | USA | 600 | Caregivers | 2 and 3 years | Dietary and energy intake |
| 237 | Maternal educational level and preschool children's consumption of high-calorie snacks and sugar-containing beverages: Mediation by the family food environment | Wijtzes, 2013 | Cross-sectional | Netherlands | 2814 | Mothers | 4 years | High-calorie snack and sugar-sweetened beverage intake |
| 238 | Nutrition-Education Program Improves Preschoolers’ At-Home Diet: A Group Randomized Trial | Williams, 2014 | Intervention (RCT) | USA | 1143 | Parents | NR | Fruit and vegetable, and low-fat milk intake |
| 239 | Increasing Fruit and Vegetable Consumption among Preschoolers: Evaluation of Color Me Healthy | Witt, 2012 | Intervention | USA | 263 | Children | 4 and 5 years | Fruit and vegetable intake |
| 240 | Characteristics of the home food environment that mediate immediate and sustained increases in child fruit and vegetable consumption: mediation analysis from the Healthy Habits cluster randomised controlled trial | Wyse, 2015 | Intervention (cluster RCT) | Australia | 326 | Parents | 3-5 years | Fruit and vegetable intake |
| 241 | Associations between characteristics of the home food environment and fruit and vegetable intake in preschool children: A cross-sectional study | Wyse, 2011 | Cross-sectional | Australia | 396 | Parents | 3-5 years | Fruit and vegetable intake |
| 242 | A cluster randomized controlled trial of a telephone-based parent intervention to increase preschoolers’ fruit and vegetable consumption | Wyse, 2012 | Intervention (cluster randomised trial) | Australia | 394 | Parents | 3-5 years | Fruit and vegetable intake |
| 243 | Parenting style and dietary behaviour of young children. Findings from the Healthy Beginnings Trial | Xu, 2013 | Cross-sectional | Australia | 242 | Mothers | 2 years | Fruit and vegetable, sugar sweetened beverage, and snack intake |
| 244 | Snacking frequency and dietary intake in toddlers and preschool children | Xue, 2019 | Cross-sectional | USA | 1186 | Caregivers | 1-6 years | Dietary intake and snacking frequency |
| 245 | Impact of mothers’ negative affectivity, parental locus of control and child-feeding practices on dietary patterns of 3-year-old children: The MoBa Cohort Study | Ystrom, 2012 | Cross-sectional | Norway | 14,122 | Mothers | 3 years | Dietary patterns |
| 246 | Is repeated exposure the holy grail for increasing children's vegetable intake? Lessons learned from a Dutch childcare intervention using various vegetable preparations? | Zeinstra, 2017 | Intervention | Netherlands | 250 | Children | 1-4 years | Vegetable intake |
